# Suppressor with morphogenetic effect on genitalia 7 (SMG7) drives hepatocellular carcinoma progression through context-dependent nonsense-mediated decay (NMD) dysregulation

**DOI:** 10.1186/s43556-026-00577-3

**Published:** 2026-10-12

**Authors:** Betsaida Ojeda-Pérez, Víctor J. Fernández-Ramírez, María I. Pozo-Relaño, Belén Gracia-Herencia, Izabela Hawro, Jose Córdoba-Chacón, Raul M. Luque, Manuel L. Rodriguez-Peralvarez, Juan L. López-Cánovas, Manuel D. Gahete

**Affiliations:** 1 Department of Cell Biology, Physiology and Immunology, University of Cordoba, Cordoba, Spain; 2 Maimonides Institute of Biomedical Research of Cordoba (IMIBIC), Cordoba, Spain; 3 Reina Sofia University Hospital (HURS), Cordoba, Spain; 4 CIBER Physiopathology of Obesity and Nutrition (CIBERobn), Madrid, Spain; 5 Department of Medicine, University of Illinois at Chicago, Chicago, IL USA; 6 Department of Hepatology and Liver Transplantation, Reina Sofía University Hospital, Córdoba, Spain; 7 CIBER Hepatic and Digestive Diseases (CIBERehd), Madrid, Spain

**Keywords:** Nonsense-mediated decay (NMD), HCC, MASLD, SMG7, UPF1, ZBP1

## Abstract

Metabolic dysfunction-associated steatotic liver disease (MASLD) is a major risk factor for hepatocellular carcinoma (HCC), yet the molecular mechanisms linking metabolic stress to tumor progression remain poorly defined. RNA surveillance pathways, including nonsense-mediated decay (NMD), may represent a relevant but unexplored regulatory layer in this context. In this study, we analyzed the expression of NMD components across multiple human HCC and MASLD cohorts, identifying suppressor with morphogenetic effect on genitalia 7 (SMG7) as a key regulator of NMD associated with HCC progression. In vitro functional assays, NMD target expression, transcriptomic profiling, and weighted gene co-expression network analysis (WGCNA) were used to identify downstream pathways and mediators. More than 40% of NMD components were dysregulated in HCC. Among them, SMG7 was consistently upregulated across HCC and MASLD cohorts, induced by lipotoxic stress, and identified as a hub gene in HCC transcriptional networks. SMG7 overexpression promoted proliferation, migration, and clonogenicity, whereas its silencing reduced tumor aggressiveness in a context-dependent manner inversely associated with UPF1 levels. Combined depletion of SMG7 and UPF1 suppressed aggressive traits in vitro and limited tumor growth in vivo. Transcriptomic and network analyses identified Z-DNA-binding protein 1 (ZBP1) as a downstream mediator of SMG7 dysregulation in SNU-387 HCC cells, a finding further supported by functional rescue experiments. These results identify the SMG7–ZBP1 axis as a metabolic stress-responsive pathway linking NMD dysfunction to liver tumor progression and highlight NMD modulation as a therapeutic strategy to restrain tumor aggressiveness while enhancing tumor immunogenicity.

**Supplementary Information:**

The online version contains supplementary material available at https://doi.org/10.1186/s43556-026-00577-3.

## Introduction

Hepatocellular carcinoma (HCC) is the most common primary liver cancer and a leading cause of cancer-related mortality worldwide [1–4]. Although historically linked to chronic viral hepatitis, HCC etiology is shifting toward non-viral drivers such as alcohol consumption and obesity-related metabolic dysfunction-associated steatotic liver disease (MASLD) [3, 4]. Despite therapeutic advances, including immunotherapy, early diagnosis remains challenging and treatment resistance is common [4–7]. Increasing evidence highlights dysregulated RNA metabolism, particularly altered splicing and non-coding RNA expression, as a key contributor to HCC development, especially during the transition from MASLD [8–11].

To maintain transcriptome integrity, cells rely on RNA surveillance pathways such as nonsense-mediated decay (NMD). NMD controls gene expression by promoting the degradation of selected mRNAs, including those arising from alternative splicing, but also a substantial fraction of physiologically normal transcripts [12]. NMD primarily acts on RNAs containing premature termination codons (PTC) more than 50–55 nucleotides upstream of an exon–exon junction, as well as on RNAs with long 3′ untranslated regions (UTRs), which often result from alternative splicing events [13]. Several factors participate in the mRNA decay process, acting at different stages. In the initial step, the exon junction complex (EJC) facilitates mRNA target export and recognition, and is composed mainly of RBM8A, eIF4A3, MAGOH, UPF2, and UPF3 [12, 13]. Subsequent steps are triggered by the formation of the suppressor with morphogenetic effect on genitalia 1 (SMG1)–UPF1–eRF1–eRF3 (SURF) complex, together with SMG8 and SMG9, which regulate SMG1 kinase activity [13–15]. The SURF complex interacts with the EJC to form the decay-inducing (DECID) complex. This regulation is essential for UPF1 phosphorylation by SMG1 and the subsequent recruitment of decay factors, including SMG6 and the SMG5–SMG7 heterodimer, leading to mRNA degradation through deadenylation, decapping, and exonucleolytic cleavage [13, 16]. Thus, NMD can also occur through EJC-independent mechanisms via long-3'UTR-mediated UPF1 recruitment [13].

Through this regulatory capacity, NMD influences diverse biological processes, including stress responses, immune regulation, and cell fate decisions [12, 13], and its dysregulation has been linked to cancer [12], including HCC, where it has been studied mainly at the level of individual components, with no comprehensive pathway-wide analysis [12, 14, 17–21].

In this context, we hypothesized that dysregulated NMD factor expression is associated with HCC progression by altering gene expression and mRNA metabolism in a context-dependent manner. To address this, we performed a global analysis of 22 NMD components across five HCC and two MASLD cohorts, identifying SMG7 as consistently upregulated and induced by palmitate. Functional assays showed that SMG7 promotes tumor aggressiveness, with its silencing effects dependent on opposing feedback regulation of UPF1. Dual depletion of SMG7 and UPF1 significantly reduced tumorigenic behavior in vitro and in vivo, while activating nucleic acid sensing and inflammatory cell-death pathways, with ZBP1 as a potential downstream effector. Overall, these findings indicate that targeting NMD through SMG7 and UPF1 may both limit tumor growth and enhance antitumor immune-related responses in HCC.

## Results

### NMD factor profiling reveals SMG7 overexpression as a common hallmark in MASLD-MASH-HCC progression

**Fig. 1:**
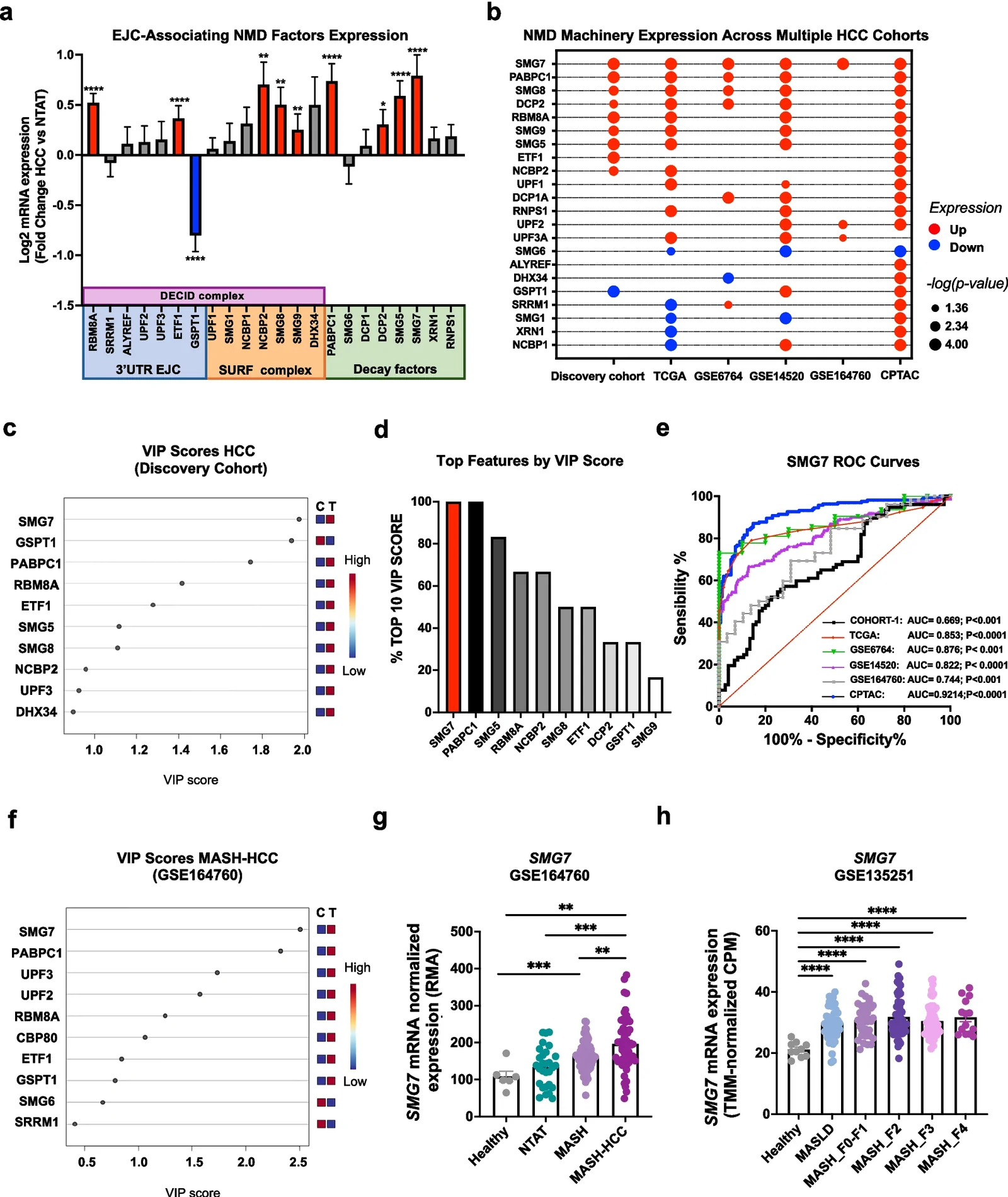
Altered expression of NMD components in HCC identifies SMG7 as the most consistently overexpressed factor across HCC and MASLD–MASH stages. **a** NMD machinery expression in the Discovery cohort. Expression values correspond to mRNA copy numbers normalized to the normalization factor (NF) and are shown as log₂ fold changes between HCC and NTAT. **b** NMD machinery expression across independent HCC cohorts: Discovery cohort (qPCR, mRNA), TCGA, (RNA-seq, transcriptome); GSE6764, GSE14520 and GSE164760 (microarray, transcriptomics), and CPTAC (mass spectrometry-based proteomics). Bubble size represents the significance (*p-value)* of the differences between HCC and control tissues, while colors indicate fold-change direction. **c** Top 10 factors ranked by variable of importance in projection (VIP) score of the 22 NMD factors analyzed in the discovery cohort comparing tumor (T) and NTAT samples (C). **d** Percentage of the cohorts in which the most consistently dysregulated factors identified in Discovery cohort ranked among the top 10 VIP scores. **e** ROC analysis of SMG7 in the Discovery cohort and five independent validation cohorts. Area under the curve (AUC) values and corresponding *p* values (P) are shown. **f** Top 10 VIP scores of the 22 NMD factors analyzed in GSE164760 comparing MASH-HCC (T) vs NTAT (C) samples. **g** SMG7 mRNA expression in healthy, NTAT, MASH, and MASH-HCC samples from GSE164760, shown as Robust Multi-array Average (RMA)-normalized expression values. **h** SMG7 mRNA expression in healthy, MASLD, and MASH samples with varying fibrosis stages (F1–F4) from GSE135251, shown as Trimmed Mean of M-values (TMM)-normalized counts per million (CPM). In panels **g** and** h**, data are presented as the mean ± standard error of the mean (SEM). Asterisks indicate statistically significant differences (*p < 0.05; **p < 0.01; ***p < 0.001, ****p < 0.0001)

The first aim of this study was to investigate the role of key factors involved in different steps of the NMD pathway, including components of the DECID complex (EJC and SURF) and mRNA decay factors, in HCC and preceding disease stages. We first analyzed the mRNA expression of the main NMD components in 86 paired HCC and non-tumoral adjacent tissue (NTAT) samples from the discovery cohort (Fig. 1a and Table S1). Transcriptional findings were validated in GSE6764 (10 normal liver vs. 35 HCC), GSE14520 (220 normal liver vs. 225 HCC), and GSE164760 (53 MASH-HCCs vs. 29 adjacent non-tumor MASH liver samples and 74 MASH samples vs. 6 healthy livers as a control) (Fig. 1b), and at the protein level using data from 159 paired HBV-related HCC and adjacent liver tissues through the National Cancer Institute’s Clinical Proteomic Tumor Analysis Consortium (CPTAC) portal (PDC000199). Detailed information on cohort composition and data sources is provided in the “Patients and samples” section of the Supplementary Material and Methods; demographic and clinical characteristics of the discovery cohort are provided in Table S1.

The analysis revealed that the expression of 45% of these components (10 out of 22) was significantly altered (most being upregulated) at the mRNA level in the discovery cohort (Fig. 1a). This dysregulation pattern was further validated across external HCC datasets at the mRNA or protein levels (Fig. 1b). Among them, SMG7 was the most consistently overexpressed factor, followed by PABPC1, SMG8, DCP2, RBM8A, SMG9 and SMG5 (Fig. 1b). Moreover, SMG7 ranked among the top 10 variables according to variable importance in projection (VIP) scores, contributing most strongly to the separation between groups in the discovery cohort (Fig. 1c). Notably, among all the altered factors in the discovery cohort, SMG7, together with PABPC1, was consistently identified within the top 10 VIP-ranked variables across all cohorts analyzed (Fig. 1d). In contrast, SMG8, DCP2, RBM8A, SMG9 and SMG5 appeared in the top 10 only in a subset of the analyzed cohorts. Receiver operating characteristic (ROC) analysis further confirmed the ability of SMG7 expression to discriminate between control and tumor samples, yielding an area under the curve (AUC) values of 0.669 in the discovery cohort and ranging from 0.744 to 0.9214 in the external cohorts (Fig. 1e). AUC values closer to 1 indicate better discriminatory performance.

Among the NMD components analyzed, *SMG7*, *UPF2* and *UPF3* showed altered expression in the GSE164760 dataset, the only cohort with well-characterized metabolic dysfunction-associated steatohepatitis (MASH)-derived HCC (Fig. 1b). Indeed, *SMG7*, *UPF2* and *UPF3* were significantly overexpressed in MASH-derived HCC samples (GSE164760 cohort, Fig. 1b), with *SMG7* showing the greatest alteration and ranking first according to its VIP score (Fig. 1f). Particularly, *SMG7* expression was significantly higher in MASH-HCC samples compared to healthy liver, NTAT, and MASH tissues (Fig. 1g). *SMG7* was also upregulated in MASH samples from an additional external cohort (GSE135251, with 206 NAFLD cases and 10 controls), and its expression increased progressively from MASLD to advanced MASH stages, according to fibrosis scores (Fig. 1h).

To further investigate the relationship between *SMG7* expression and MASLD stages, we employed an in vitro model of steatosis, early MASH and late MASH, generated by THLE-2 cells treated with oleic acid (OA), OA plus palmitic acid (PA) (2:1 ratio) or PA, respectively (Fig. S1a-e), as previously reported [22–24]. Inflammatory markers were assessed to validate the effects of each treatment (Fig. S1b-d). As expected, PA exposure significantly induced *NLRP3* and *IL1B* (Fig. S1b, c), while treatment or co-treatment with oleic acid (OA) mitigated this response [25] and tended to increase the expression of the hepatoprotective factor *MIF* [26] (Fig. S1d). Under these conditions, *SMG7* mRNA levels were significantly higher after PA exposure than after OA and tended to be elevated compared to OA:PA treatment (Fig. S1e). Consistent with the increased lipid droplet accumulation detected by BODIPY staining, PA treatment for 24 h in mouse primary hepatocytes (MPH) significantly induced *Smg7* mRNA expression (Fig. S1f). Accordingly, SMG7 protein levels differed among the cell lines analyzed (Fig. S1g). Particularly, SMG7 protein expression was comparatively lower in non-tumoral THLE-2 cells than in HCC cell lines, with readily detectable levels in Hep3B cells and further increased in SNU-387 cells. Consistently, Western blot densitometry (SMG7/β-tubulin ratio) revealed approximately 50% higher normalized SMG7 protein levels in SNU-387 than in Hep3B cells (Fig. S1g).

Collectively, these findings identify SMG7 as the most consistently dysregulated NMD factor across HCC cohorts, with progressive upregulation during MASLD/MASH-associated hepatocarcinogenesis, supporting its potential as a biomarker and candidate mediator of liver cancer progression.

### SMG7 overexpression enhances the aggressiveness of HCC cells

**Fig. 2:**
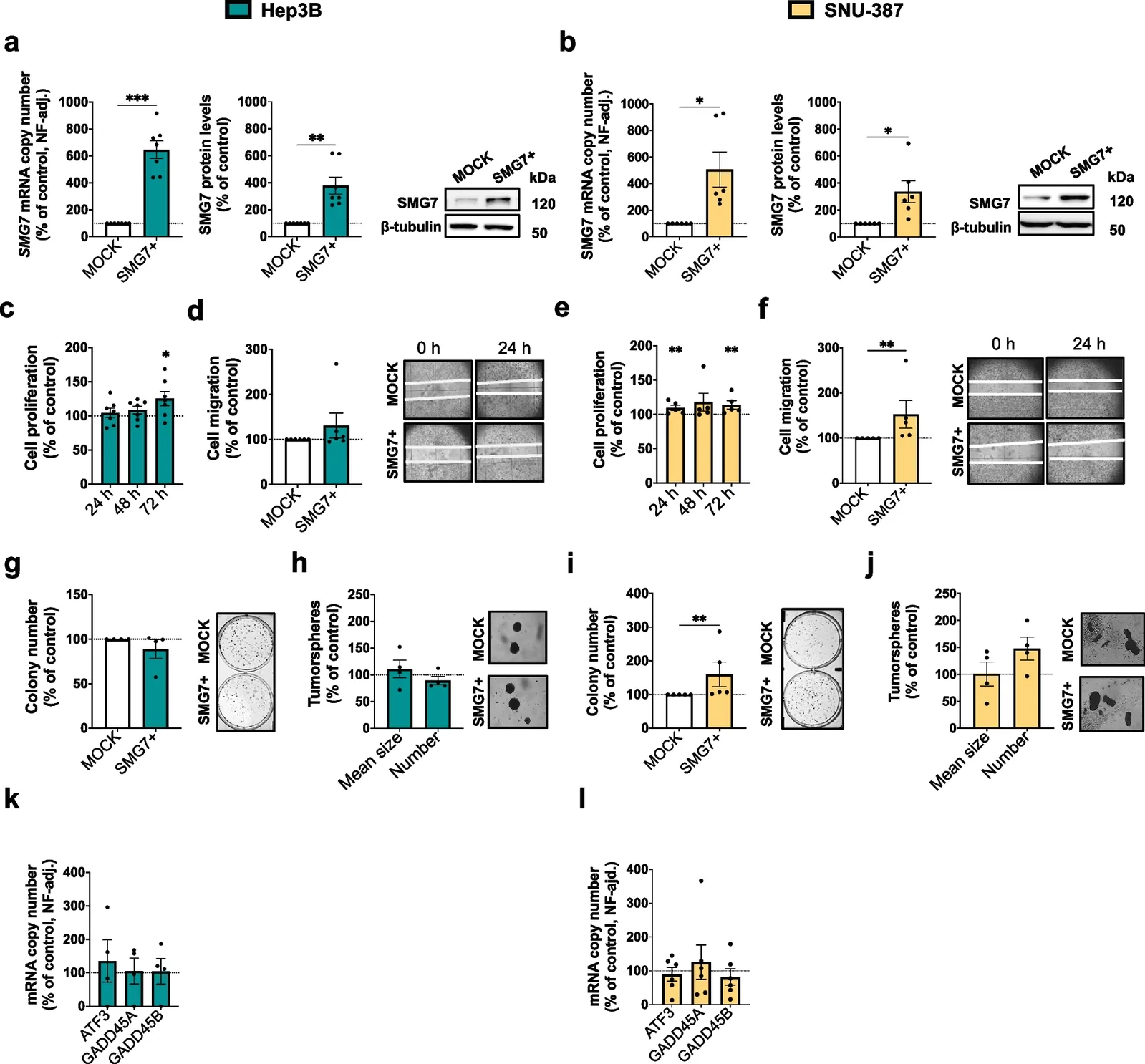
*SMG7* overexpression contributes to aggressive phenotypes in a cell line-dependent manner in HCC models. **a, b** Validation of *SMG7* overexpression (SMG7 +) at mRNA and protein levels in Hep3B (**a**) and SNU-387 (**b**). **c-j** Cellular proliferation (**c** and **e**), cell migration (**d** and **f**), colony formation (**g** and **i**), and tumorsphere formation (**h** and **j**) of Hep3B and SNU-387, respectively, after *SMG7* overexpression compared with the control (mock). **k-l** mRNA expression levels of *ATF3*, *GADD45A* and *GADD45B* in SMG7 + in Hep3B (**k**) and SNU-387 (**l**) in comparison with mock. The sample size included at least three biological replicates. Data is represented as mean ± SEM. mRNA expression is presented as normalized copy numbers and expressed as a percentage of the control (set to 100%). Protein levels are normalized to β-tubulin and expressed as % of control. Asterisks (*p < 0.05; **p < 0.01; ***p < 0.001) indicate statistically significant differences. mRNA expression is presented as normalized copy numbers and expressed as a percentage of the control (set to 100%)

In order to functionally demonstrate the role of SMG7, its overexpression was induced and confirmed at the mRNA and protein levels in HCC cell lines (Fig. 2a, b). *SMG7* overexpression increased cell proliferation in both lines and migration capacity in SNU-387 (Fig. 2c-f). *SMG7* overexpression also promoted colony formation in SNU-387, but not in Hep3B cells, while it had no statistically significant effects on tumorsphere formation (Fig. 2g-j). These findings indicate that *SMG7* overexpression enhances aggressive properties especially in the mesenchymal-like SNU-387 cells, suggesting that SMG7 may contribute to the proliferative and invasive potential of these cells.

To assess the effect of *SMG7* overexpression on NMD activity, the relative expression levels of three natural NMD targets described previously [27, 28] (*ATF3*, *GADD45A*, and *GADD45B*) were measured in Hep3B (Fig. 2k) and SNU-387 cells (Fig. 2l). The results showed that elevated SMG7 levels did not significantly alter the expression of these targets in any cell line. However, *SMG7*-overexpressing Hep3B cells showed reduced protein levels of UPF1 (the key effector of the NMD pathway [13]) (Fig. S2a), with a non-significant reduction in UPF1 phosphorylation (Fig. S2b). In contrast, *SMG7*-overexpressing SNU-387 cells exhibited increased *UPF1* mRNA levels, but no corresponding changes at the protein level (Fig. S2c). Upon *SMG7* overexpression, UPF1 phosphorylation tended to be higher in SNU-387 cells (Fig. S2d), indicating that the consequences of *SMG7* overexpression in terms of UPF1 expression and activity may be cell-type dependent.

### *SMG7* depletion elevates UPF1 and inhibits SNU-387 cell growth and migration

**Fig. 3:**
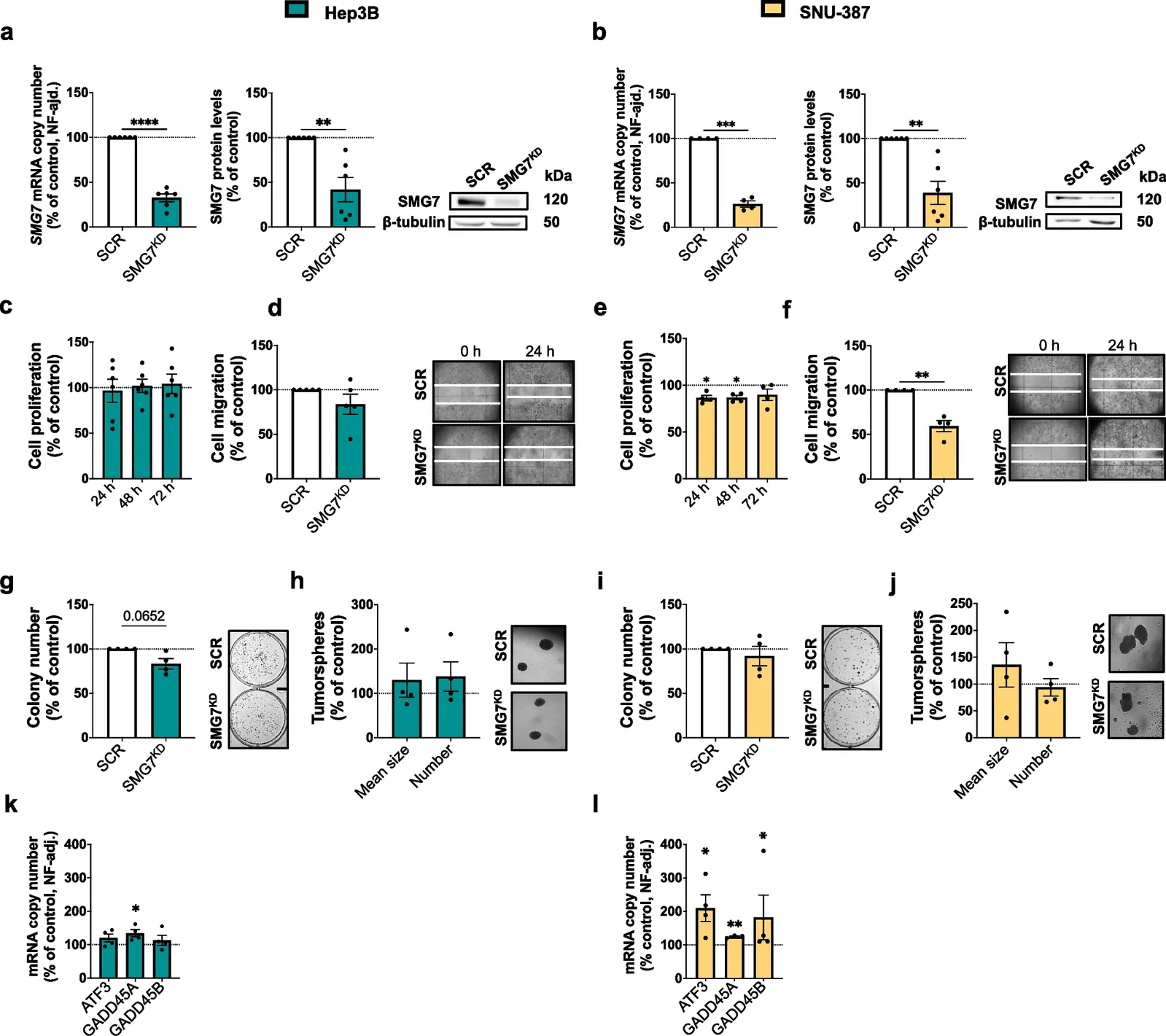
*SMG7* knockdown attenuates tumoral capacity in SNU-387 HCC cells. **a**,** b** Validation of *SMG7* knockdown (SMG7^KD^) in Hep3B (**a**) and SNU-387 (**b**) at mRNA and protein levels. **c-j **Cellular proliferation (**c** and **e**), cell migration (**d** and **f**), colony formation (**g** and **i**), and tumorsphere formation (**h** and **j**) of Hep3B and SNU-387, respectively, after *SMG7* silencing comparing with control (SCR). **k-l** mRNA expression levels of *ATF3*, *GADD45A* and *GADD45B* in SMG7^KD^ in Hep3B (**k**) and SNU-387 (**l**) in compared with the control (SCR). The sample size included at least three biological replicates. Data is represented as mean ± SEM. mRNA expression is presented as normalized copy numbers and expressed as a percentage of the control (set to 100%). Protein levels are normalized to β-tubulin and expressed as % of control. Asterisks (*p < 0.05; **p < 0.01; ***p < 0.001) indicate statistically significant differences. The values displayed on the graphs correspond to *p*-values

To study the effect of SMG7 loss-of-function in HCC cell lines, transient silencing of *SMG7* in Hep3B and SNU-387 cell lines was performed. This resulted in a 55–67% average reduction in both mRNA and protein levels after 24 h (Fig. 3a, b). In Hep3B cells, *SMG7* knockdown had no effect on proliferation and migration (Fig. 3c, d); however, it significantly impaired proliferation and migration in SNU-387 (Fig. 3e, f). Additionally, the Hep3B cell line showed a trend toward reduced colony formation (Fig. 3g), but this effect on colony number was not observed in SNU-387 cells (Fig. 3i). Finally, tumorsphere formation was not altered in either Hep3B or SNU-387 cells (Fig. 3h, j). This further supports the increased sensitivity of mesenchymal-like SNU-387 cells to *SMG7* modulation.

Expression of NMD target genes was also evaluated after *SMG7* silencing. Hep3B exhibited a slight upregulation of all three genes (to an average between 113–134% of scramble control levels), with only *GADD45A* being significantly elevated (Fig. 3k). On the other hand, SNU-387 showed a significant upregulation of *ATF3, GADD45A* and *GADD45B* (Fig. 3l). Interestingly, *SMG7* suppression led to UPF1 upregulation in both cell lines. However, p-UPF1 levels tended to be elevated in Hep3B but not in SNU-387 (Fig. S2e-h), reinforcing the cell type-dependent consequences of SMG7 levels within the HCC context.

### *SMG7* and *UPF1* co-silencing reduces HCC cell aggressiveness in vitro and in vivo

To investigate the co-regulatory activity between UPF1 and SMG7, *UPF1* knockdown was first performed. Similar effects to those observed with *SMG7* silencing were observed (Fig. S3). In Hep3B and SNU-387, *UPF1* depletion, validated at mRNA and protein levels, also induced SMG7 overexpression, suggesting a potential regulatory feedback loop (Fig. S3a-d). Interestingly, *UPF1* silencing had no significant impact on proliferation, colony formation, migration, or tumorsphere development in Hep3B; however, reduced UPF1 levels in SNU-387 cells impaired both proliferation and migration (Fig. S3e-l). In Hep3B cells, *ATF3* mRNA was upregulated and the amount of p-UPF1 protein was lower when *UPF1* was silenced (Fig. S3m, n). However, *ATF3*, *GADD45A* and *GADD45B* levels were not significantly higher in SNU-387 and p-UPF1 was not significantly reduced under *UPF1* depletion (Fig. S3o, p). These results indicate strong compensatory feedback between SMG7 and UPF1 to maintain NMD activity when the levels of SMG7 or UPF1 are reduced.

**Fig. 4:**
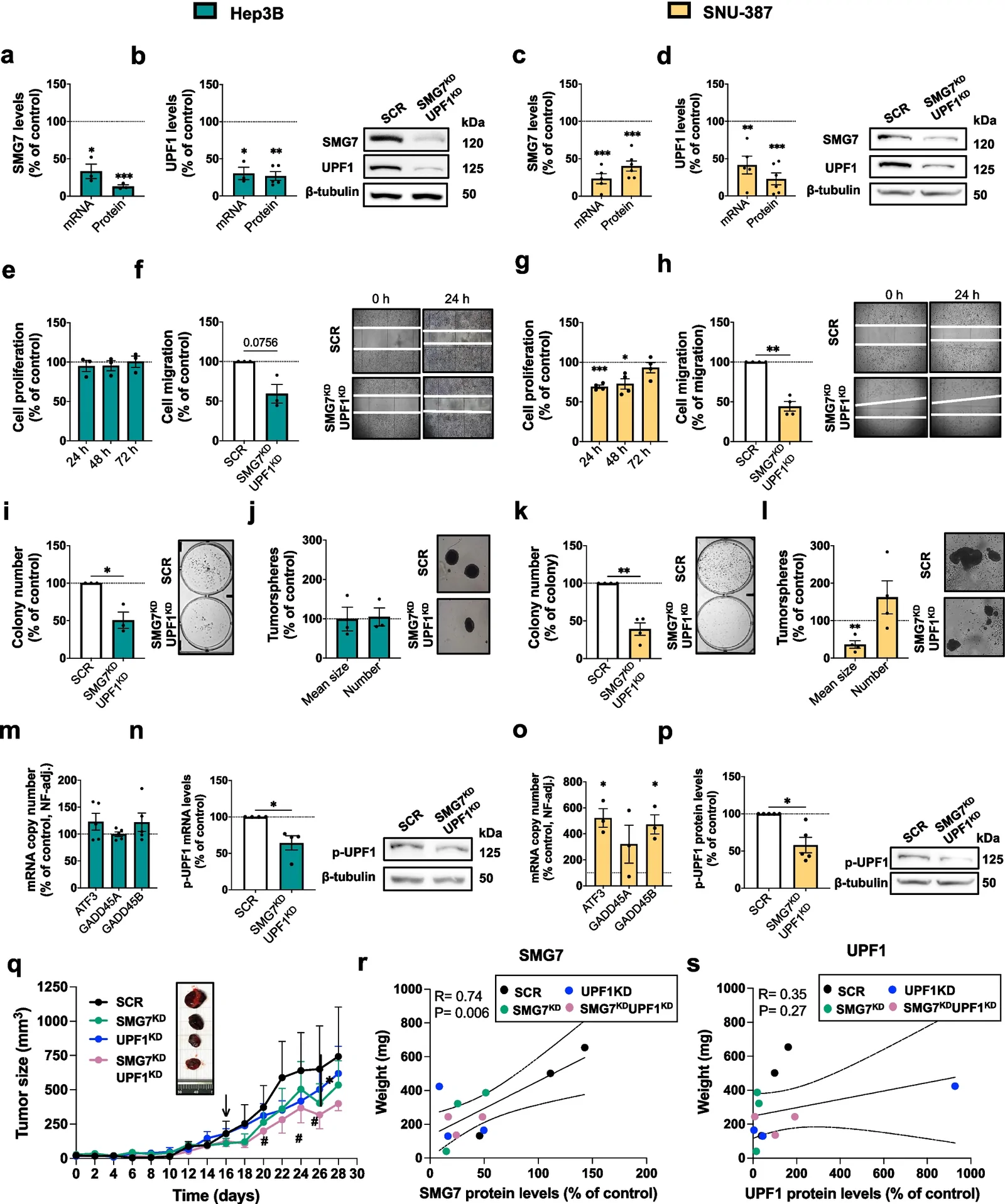
*SMG7* and *UPF1* co-silencing affects tumor features in HCC cell lines. **a-d **Validation of *SMG7* and *UPF1* co-silencing (SMG7^KD^UPF1^KD^) in Hep3B (**a** and **b**) and SNU-387 (**c** and **d**) at mRNA or protein levels. **e-l** Cellular proliferation, cell migration, colony number and tumorsphere formation of Hep3B and SNU-387 SMG7^KD^UPF1^KD^ comparing with control (SCR). **m-p **Analysis of NMD efficiency by measuring NMD target mRNA levels and of p-UPF1 protein levels in Hep3B (**m** and **n**) and SNU-387 (**o** and **p**). **q** Tumor volume (mm^3^) of SMG7^KD^, UPF1^KD^ and SMG7^KD^UPF1^KD^ tumors vs. control (SCR) during the experiment of xenograft model (28 days). **r** Correlation between tumor weight and SMG7 protein levels in SMG7^KD^, UPF1^KD^ and SMG7^KD^UPF1^KD^, and SCR tumors. **s** Correlation between tumor weight and UPF1 protein levels on SMG7^KD^, UPF1^KD^ and SMG7^KD^UPF1^KD^, and SCR tumors. The sample size included at least three biological replicates. Data is represented as mean ± SEM. mRNA expression is presented as normalized copy numbers and expressed as a percentage of the control (set to 100%). Protein levels are normalized to β-tubulin (**a-d**, **r**, and **s**) and expressed as % of control (**a**-**d**). Asterisks (*p < 0.05; **p < 0.01; ***p < 0.001) indicate statistically significant differences. The values displayed on the graphs correspond to *p*-values. In panel **q**, the arrow indicates the time of siRNA injection, asterisk (*p < 0.05) denotes significance for the column factor based on two-way ANOVA, while hash symbols (#*p* < 0.05) indicate significant differences at individual time points determined by Fisher’s LSD test

Building on our findings and the proposed co-regulatory role of UPF1 and SMG7, we next performed simultaneous silencing of both genes to evaluate whether their combined knockdown exerts additional effects on HCC cell lines. Effective knockdown of both genes (*SMG7* and *UPF1*) was confirmed at the mRNA and protein levels (Fig. 4a-d) in both cell lines.

Functional assays in Hep3B cells revealed that co-silencing resulted in a reduced migratory tendency and a significant decrease in colony formation, although proliferation and tumorsphere number or size remained unchanged. In SNU-387 cells, the combined knockdown not only impaired proliferation at 24 and 48 h, but also significantly reduced migration, colony formation, and tumorsphere size, indicating a more pronounced anti-tumor effect of dual *SMG7* and *UPF1* silencing in HCC cell lines (Fig. 4e-l).

Direct comparison of the three knockdown conditions showed no significant differences in proliferation among conditions in Hep3B cells (Fig. S4a), whereas differences between *UPF1* knockdown and co-silencing emerged at 24 and 48 h in SNU-387 cells (Fig. S4b). Migration did not differ significantly among the knockdown conditions in Hep3B cells (Fig. S4c). Although co-silencing reduced colony formation in Hep3B cells, a significant difference was observed only relative to *UPF1* knockdown (Fig. S4d). In SNU-387 cells, migration was similarly affected across all knockdown conditions (Fig. S4e), whereas co-silencing reduced colony formation, with a significant difference observed only relative to *SMG7* knockdown (Fig. S4f). Neither tumorsphere number nor tumorsphere size differed significantly among the knockdown conditions in Hep3B cells (Fig. S4g, h). In SNU-387 cells, tumorsphere number was also unaffected (Fig. S4i), whereas co-silencing produced a greater reduction in tumorsphere size than either single knockdown, with significant differences only relative to *UPF1*-silenced cells (Fig. S4j).

Unexpectedly, simultaneous silencing of *SMG7* and *UPF1* did not affect the expression of NMD target genes in Hep3B cells, despite causing a significant reduction in p-UPF1 levels, (Fig. 4m, n). In contrast, in SNU-387 cells, the combined knockdown resulted in the upregulation of NMD targets *ATF3* and *GADD45B* and a reduction of p-UPF1 (Fig. 4o, p).

To corroborate the antitumor effect of *SMG7* and *UPF1* co-silencing, a preclinical assay using a xenograft model was conducted. As expected, a significant decrease in tumor growth was observed from day 22 when both *SMG7* and *UPF1* were co-silenced, while no differences were observed with SMG7^KD^ or UPF1^KD^ alone (Fig. 4q). In addition, only combined silencing showed a trend toward reduced tumor weight compared with controls (Fig. S5a). Effective *SMG7* and *UPF1* knockdown was confirmed in the different experimental groups at the protein levels, although UPF1 reduction did not reach statistical significance due to the limited sample size (*n* = 3 mice per group) (Fig. S5b, c). In addition, SMG7 protein expression, but not UPF1, was positively correlated with tumor weight under all conditions (single and double silencing) (Fig. 4r, s). This suggests a potential role for the combined reduction of *SMG7* and *UPF1* in hindering tumor progression in HCC, confirming that *SMG7* or *UPF1* modulation alone would be less effective.

### *SMG7* depletion in SNU-387 cells enhances nucleic acid sensing and inflammatory cell death pathways

**Fig. 5:**
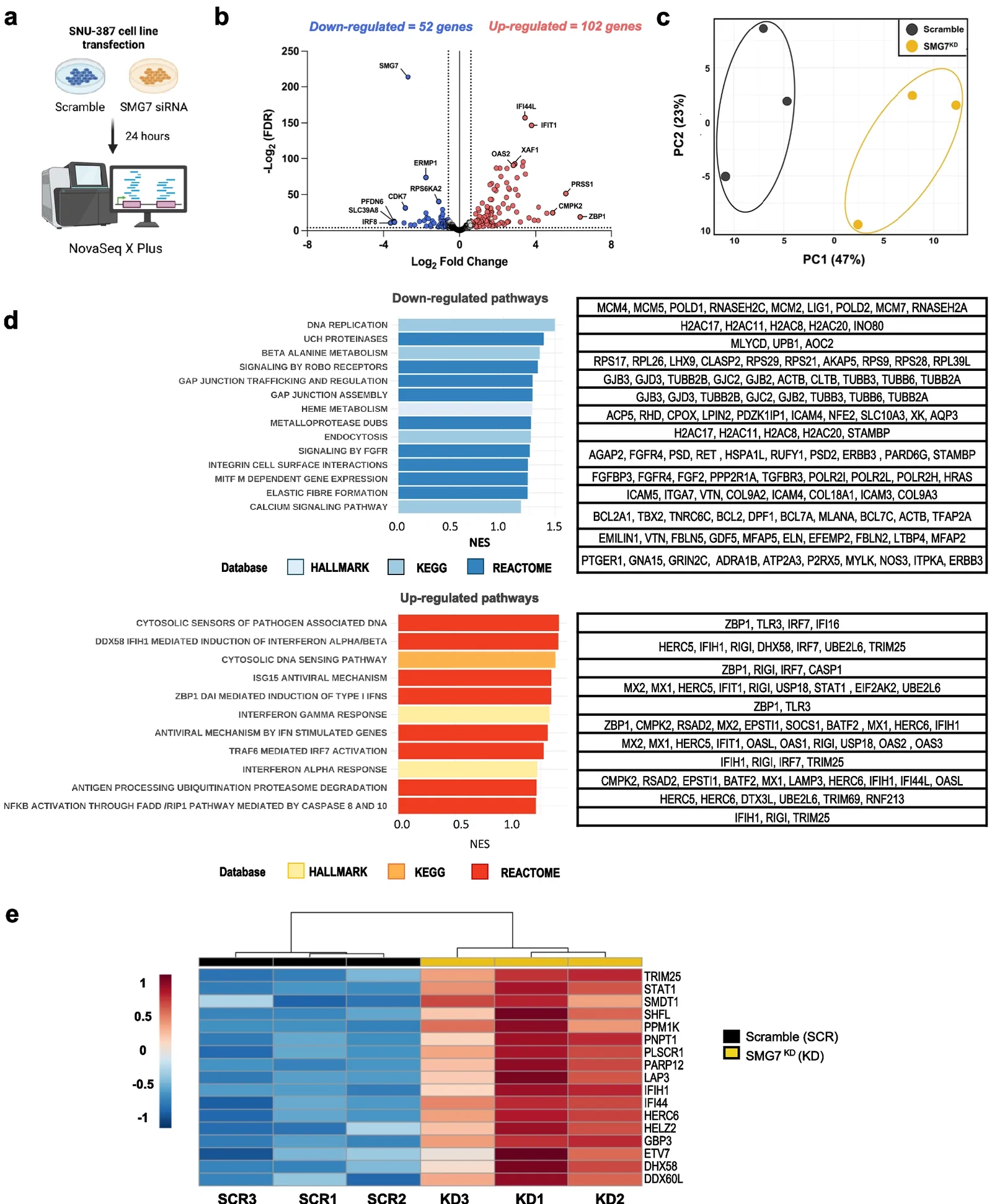
RNA-seq reveals enriched cytosolic nucleic acid sensing and inflammatory pathways in SNU-387 SMG7^KD^ cells. **a** Schematic representation of the experimental design. Created with BioRender.com (agreement number: GQ2A1Q028N). **b** Volcano plot showing significantly downregulated (blue, log₂ fold change ≤ –0.58) and upregulated (red, log₂ fold change ≥ 0.58) genes in SMG7^KD^ cells (*n* = 3). **c** Principal component analysis (PCA) showing distinct clustering of control (scramble) and SMG7^KD^ samples. **d** Bar plot of Gene Set Enrichment Analysis (GSEA) results showing enriched upregulated (top) and downregulated (bottom) pathways from the Hallmark, KEGG, and Reactome databases. Pathways are ranked by Normalized Enrichment Score (NES) (NOM *p*-value < 0.001), with the most representative genes indicated by their highest Running Enrichment Score (Running ES). **e** Heat map showing the expression profiles of the 17 candidate NMD-regulated genes that were significantly upregulated following SMG7 depletion. Hierarchical clustering was performed using Euclidean distance and Ward's method (ward. D)

To investigate the role of SMG7 in HCC, we performed transcriptomic profiling by RNA-seq in SNU-387 cells following *SMG7* knockdown (Fig. 5a and Supplementary Material 1). *SMG7* silencing induced a marked shift in the transcriptomic profile, with significant changes in gene expression: 102 genes were upregulated above a 1.5-fold change threshold (log_2_FC > 0.58) and 52 genes were downregulated (log₂FC < –0.58) (Fig. 5b). Among the most significantly upregulated genes *IFIT1, IFI44L, XAF1, OAS2, ZBP1, PRSS1* and *CMPK2* were identified. Conversely, the most significantly downregulated genes included *RPS6KA2, ERMP1, CDK7, SLC39A8, PFDN6* and *IRF8* (Fig. 5b). Principal component analysis (PCA) showed a clear segregation of control and SMG7-depleted samples, indicating that SMG7 depletion is a major driver of global transcriptomic variation in HCC cells (Fig. 5c).

Gene Set Enrichment Analysis of the SNU-387 SMG7^KD^ RNA-seq showed a strong enrichment of innate immune pathways, particularly those involving cytosolic nucleic acid sensing and type I interferon responses, as well as DDX58/IFIH1 signaling, ISG15 antiviral mechanisms, and NF-κB pathways (Fig. 5d). In contrast, *SMG7* silencing led to a coordinated downregulation of signaling networks implicated in intercellular communication (e.g., gap junctions, integrin interactions), extracellular matrix remodelling (including metalloprotease activity and elastic fiber formation), metabolic pathways (e.g., heme and β-alanine metabolism), and proliferative and pro-survival mechanisms such as FGFR, DNA replication, and calcium signaling (Fig. 5d). The genes shown on the right side of the pathway diagrams represent those with the highest influence based on differential expression analysis filtered by higher rank metric scores. Many of these genes were recurrent and significantly upregulated across multiple enriched pathways in SMG7^KD^ samples, including *IFIH1* and *RIGI* (present in 5 out of 11 pathways), *ZBP1* and IRF7 (4 out of 11), as well as *HERC5* and *MX1* (also 4 out of 11). In contrast, the most influential genes within pathways enriched in the control condition were more heterogeneous. Only a subset (including *H2AC17, H2AC11, H2AC8, H2AC20, GJB3, GJD3, ACTB, TUBB2B, GJC2, GJB2, TUBB3, TUBB6, TUBB2A, ICAM4*, and *VTN*) appeared in 2 out of the 14 enriched pathways. Among them, *MCM5*, *MLYCD* and *RPS29* were also significantly downregulated in *SMG7* knockdown (SMG7^KD^) samples (Fig. 5d).

Among the 102 genes upregulated following *SMG7* knockdown in SNU-387 cells, 17 fulfilled the criteria for putative NMD-regulated transcripts according to the resource NMDRHT (NMD-Regulated Human Transcriptome) database [29] (Fig. 5e). Cross-cohort analysis revealed that several of these candidates displayed consistent expression patterns in HCC, with *HERC6* and *IFI44* dysregulated in three cohorts (Table S2). These findings indicate that a subset of genes induced following SMG7 depletion corresponds to putative NMD-regulated transcripts displaying the opposite expression pattern in human HCC datasets. In contrast, validation of the remaining non-NMD target genes was less consistent across datasets, and the number of recurrent genes markedly decreased with increasingly stringent log_2_FC thresholds, indicating that most expression changes were cohort-specific (Tables S3-S8).

### ZBP1 is associated with SMG7^KD^-enriched pathways and emerges as a candidate downstream effector of SMG7/UPF1 perturbation in SNU-387

To identify candidate downstream mediators of SMG7, we analyzed the transcriptomic changes induced by *SMG7* depletion. This analysis identified *ZBP1* as the most significantly upregulated gene (log_2_FC = 6.35) and one of the most prominent genes across the enriched pathways (Fig. 5b, d). Search Tool for the Retrieval of Interacting Genes/Proteins (STRING) network analysis revealed that ZBP1 is associated with many of the proteins encoded by genes upregulated in *SMG7* knockdown samples. The confidence of these associations was high, with an average local clustering coefficient of 0.686 and protein–protein interaction (PPI) enrichment *P* < 10^–16^, indicating that these proteins are biologically linked, at least in part, within the same network (Fig. S6a). The upregulated genes are involved in multiple RNA-related and regulatory activities, including binding to both single- and double-stranded RNA, or exhibiting enzymatic functions such as 2′–5′ oligoadenylate synthetase activity, adenylyltransferase activity, and RNA helicase activity. They also participate in ubiquitin-mediated protein modification, with ubiquitin ligase and transferase activities, and have ADP-ribosyltransferase activity using NAD⁺ (Fig. S6a, b).

**Fig. 6:**
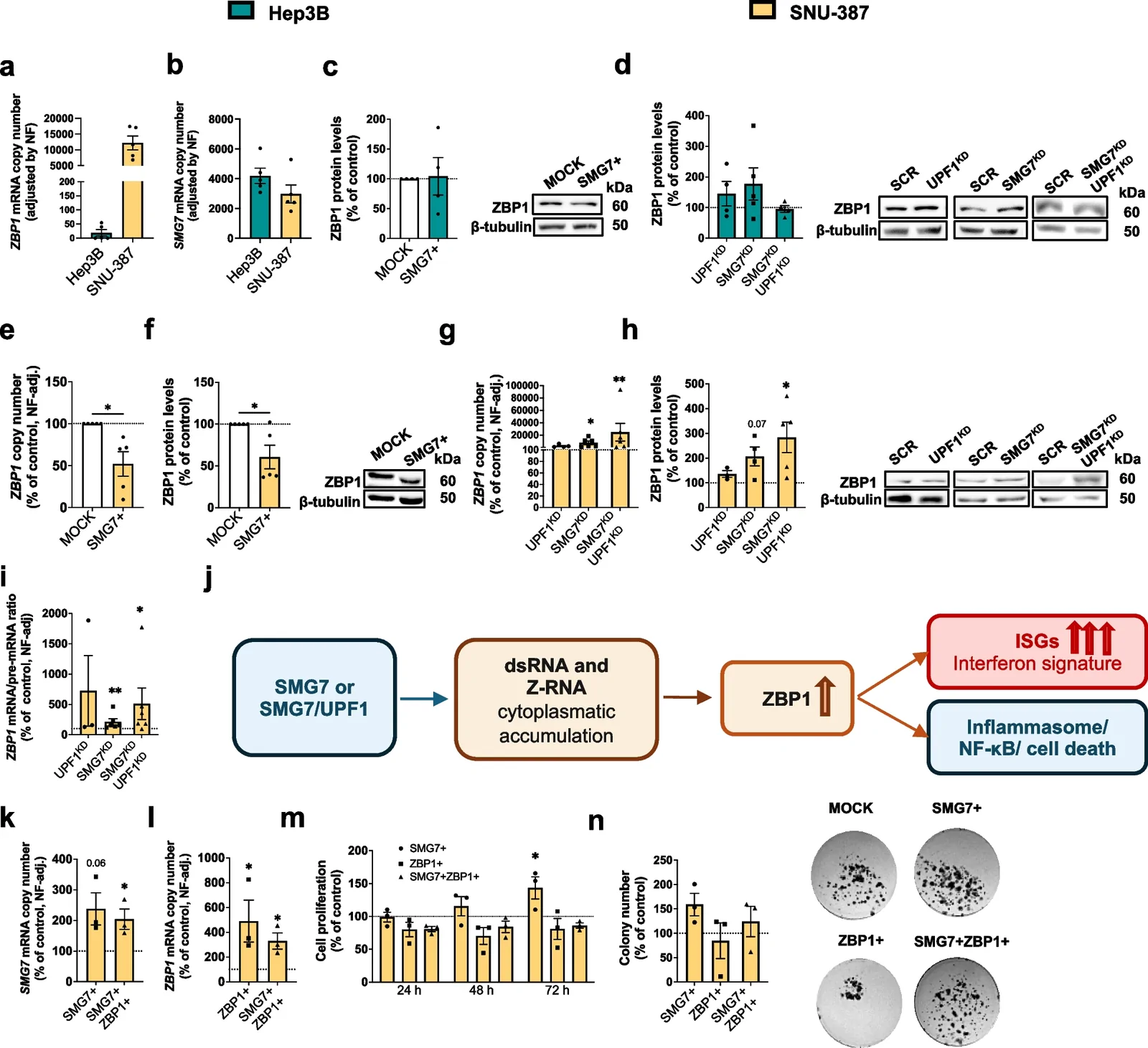
*ZBP1* is induced by *SMG7/UPF1* modulation and rescues the SMG7-overexpression phenotype in SNU-387 cells. **a** mRNA copy number normalized to the normalization factor (NF) of *SMG7* in Hep3B and SNU-387. **b** Copy number normalized by NF of *ZBP1* in Hep3B and SNU-387. **c** ZBP1 relative protein levels in Hep3B SMG7 +. **d** ZBP1 relative protein levels in Hep3B SMG7^KD^, UPF1 ^KD^ and SMG7 ^KD^/UPF1 ^KD^. **e,** **f** ZBP1 mRNA and protein levels in SNU-387 SMG7 +. **g**, **h** ZBP1 mRNA and protein levels in SNU-387 SMG7^KD^, UPF1 ^KD^ and SMG7 ^KD^/UPF1 ^KD^. **i** mRNA-to-pre-mRNA expression ratio of *ZBP1* in SNU-387 SMG7^KD^, UPF1 ^KD^ and combined SMG7 ^KD^/UPF1 ^KD^. **j** Schematic representation of the proposed mechanism by which ZBP1 acts as a downstream effector of SMG7/UPF1. **k** *SMG7* mRNA expression in SNU-387 SMG7 + and SMG7 + ZBP1 +. **l** *ZBP1* mRNA expression in SNU387 ZBP1 + and SMG7 + ZBP1 +. **m** Percentage of proliferation of SNU387 SMG7 +, ZBP1 +, and combined SMG7 + ZBP1 + relative to conttrol (mock). **n** Percentage of colony number of SNU3-87 SMG7 +, ZBP1 +, and combined SMG7 + ZBP1 + relative to control (mock). mRNA expression is presented as normalized copy numbers and expressed as a percentage of the control (set to 100%). Protein levels are normalized to β-tubulin and expressed as % of control. The sample size included at least three biological replicates. Data is represented as mean ± SEM. Asterisks (*p < 0.05; **p < 0.01; ***p < 0.001; ****p < 0.0001) indicate statistically significant differences. The values displayed on the graphs correspond to *p*-values

Remarkably, while *SMG7* mRNA expression was comparable in both HCC cell lines used herein (Fig. 6a), *ZBP1* mRNA levels were very low in Hep3B cells compared to SNU-387 cells (Fig. 6b). Nevertheless, ZBP1 protein was detectable in Hep3B cells and remained unchanged following *SMG7* overexpression or single/combined knockdown of *SMG7* and *UPF1* (Fig. 6c, d). In SNU-387 cells, ZBP1 mRNA and protein levels decreased upon *SMG7* overexpression (Fig. 6e, f) and increased following *SMG7* knockdown (Fig. 6g, h). Interestingly, *UPF1* knockdown did not significantly increase ZBP1 levels (Fig. 6g, h). Notably, co-silencing of *SMG7* and *UPF1* led to at least a threefold increase in *ZBP1* transcripts and higher ZBP1 protein levels, compared to single-gene silencing (Fig. 6g, h). Moreover, we explored the possibility that *ZBP1* transcript could be regulated post-transcriptionally by measuring the ratio between mature mRNA and pre-mRNA. The results showed that the ratio was elevated after the silencing of *SMG7* or co-silencing with *UPF1* in SNU-387 (Fig. 6i). This suggests that ZBP1 plays a role mediating the effects of SMG7 in SNU-387 cells (Fig. 6j), unlike Hep3B where other ZBP1-independent pathways could be acting.

To investigate the contribution of ZBP1 to the effects induced by SMG7, SNU-387 cells were transfected to individually overexpress *SMG7* or *ZBP1*, or to co-express both genes. Successful overexpression was confirmed at the mRNA level (Fig. 6k, l). Notably, co-expression of *SMG7* and *ZBP1* attenuated the increase in cell proliferation induced by SMG7 overexpression alone at 72 h (Fig. 6m). In addition, a similar trend was observed in colony formation assays, although the differences did not reach statistical significance (Fig. 6n).

### WGCNA identifies *SMG7* and *ZBP1* as central hub genes in HCC transcriptional modules associated with biological processes and clinical traits

**Fig. 7:**
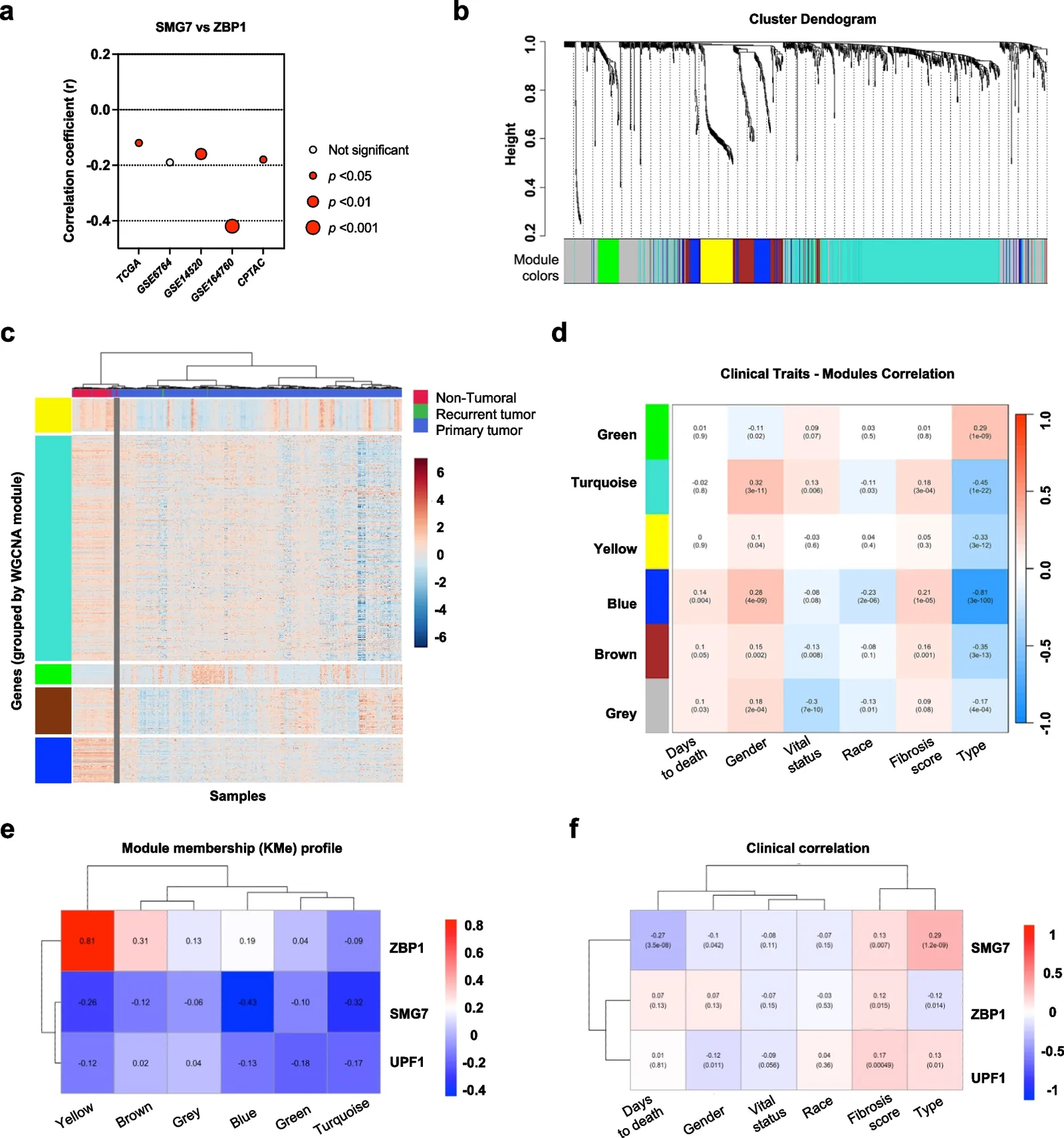
Distinct co-expression modules reflect the negative association between SMG7 and ZBP1 and link SMG7 to adverse clinical features in HCC. **a** Correlation between *SMG7* and *ZBP1* mRNA or protein expression across independent tumor samples of HCC cohorts. Dot size indicates the statistical significance level (*p*-value) of the negative correlation coefficients (R) across TCGA (371 HCC), GSE6764 (35 HCC), GSE14520 (225 HCC), GSE164760 (53 MASH-HCCs), and CPTAC (159 HCC) cohorts. **b-f** WGCNA of the TCGA-LIHC cohort (419 samples) identified gene co-expression modules associated with *SMG7*, *ZBP1*, and clinical traits. **b** Hierarchical clustering dendrogram of TCGA. Genes are clustered based on topological overlap, with the bottom color bar designating the six identified co-expression modules (Green, Turquoise, Yellow, Blue, Brown, and Grey). The grey module contains genes not assigned to any co-expression module. **c** Unsupervised hierarchical clustering and expression heatmap of module-associated genes across samples by MetaboAnalyst. The top annotation bar categorizes samples into Non-Tumoral (red), Recurrent Tumor (green), and Primary Tumor (blue). **d** Heatmap of module–trait relationships. Each cell displays the correlation coefficient and its corresponding* p*-value (in parentheses) between module eigengenes and selected clinical characteristics (Days to death, Gender, Vital status, Race, Fibrosis score, and Sample Type). **e** Module membership (Kme) profile heatmap displaying the specific correlation weights of *ZBP1*, *SMG7*, and *UPF1* across the six identified co-expression modules. **f** Gene-level clinical correlation heatmap showing the direct relationship (correlation coefficients and *p*-values) between *SMG7*, *ZBP1*, and *UPF1* expression and individual clinical traits. Color scales in (**d**), (**e**), and (**f**) denote the strength and direction of the correlations (red = positive, blue = negative)

Following in vitro functional validation, we examined the correlation between SMG7 and ZBP1 across multiple independent datasets, revealing a consistent and significant negative correlation in TCGA, GSE14520, GSE164760, and CPTAC cohorts (Fig. 7a). To understand their role within the broader HCC transcriptional landscape, we performed a Weighted Gene Co-expression Network Analysis (WGCNA) using the TCGA dataset, ensuring the inclusion of *SMG7*, *ZBP1*, and *UPF1* regardless of variance rank. Using an optimal soft-thresholding power of β = 6 (Fig. S7a, b) and an eigengene dissimilarity merge threshold of less than 0.25, hierarchical clustering classified the transcriptome into six distinct co-expression modules: turquoise, grey, blue, brown, yellow, and green (Fig. 7b). Subsequent unsupervised hierarchical clustering of samples based on module expression profiles clearly segregated HCC from non-tumoral control samples, confirming that the identified co-expression modules effectively capture disease-specific transcriptional alterations (Fig. 7c). Module-specific gene expression trajectories across samples are shown in Fig. S7c. Among all modules, the highest-ranking VIP-scored genes belonged to the blue module (Fig. S7d).

Next, we evaluated module–trait relationships to link these co-expression clusters with clinical features (Fig. 7d). Several modules demonstrated robust associations with traits such as etiology, fibrosis score, race, or survival. Notably, the blue module correlated negatively with tumoral status and positively with survival. When evaluating the specific alignment of our genes of interest within this network, *ZBP1* correlated positively with the yellow module, whereas *SMG7* correlated negatively with the blue module; *UPF1* did not show a module-specific association (Fig. 7e). Direct gene-trait correlation analysis further supported these findings: *SMG7* displayed a stronger positive correlation with sample type (*r* = 0.29) than *UPF1* (*r* = 0.13) and a significant negative correlation with days to death (*r* = − 0.27), a trajectory not significantly mirrored by *UPF1*. Conversely, *ZBP1* expression was negatively correlated with sample type (Fig. 7f).

Finally, network visualization and functional enrichment mapping using Cytoscape identified *SMG7* and *ZBP1* as potential hub genes residing within distinct network neighbourhoods (the blue and yellow modules, respectively). Both genes were topologically connected to extensive co-expression subnetworks involved in distinct pathophysiological programs, with *SMG7* nodes being heavily enriched in cellular proliferation and detoxification processes, and *ZBP1* networks strongly associated with immune responses (Fig. S8).

Taken together, these findings support a model in which SMG7 and ZBP1 occupy distinct but interconnected transcriptional programs in HCC, consistent with the negative regulation of ZBP1 by SMG7 and their opposing associations with tumor progression and immune-related processes.

## Discussion

In the past decade, growing attention has been directed toward the NMD machinery because of its significant implication in diverse pathological processes [12, 30]. NMD alterations have been linked to cancer, where this pathway may exert context-dependent tumor-suppressive or tumor-promoting roles, yet key gaps remain regarding its contribution to tumor progression and therapeutic relevance [12, 30].

Our results indicate that NMD components are markedly altered in HCC, with several NMD core factors and other genes involved in NMD consistently overexpressed in cross-cohort analyses, including SMG7, PABPC1, SMG8, DCP2, RBM8A, SMG9 and SMG5. Notably, PABPC1, DCP2, RBM8A, SMG9 and SMG5 overexpression has been linked to HCC development and unfavourable clinical outcomes [14, 21, 31–37]. Although no prior studies have implicated SMG7 in liver disease, our findings demonstrate that it is among the most consistently overexpressed NMD factors across internal and external HCC datasets. Consistently, SMG7 ranked among the top discriminative genes between HCC and control samples and showed strong diagnostic performance, supporting its potential value as a biomarker.

Chronic liver diseases (CLD) increase the risk of tumor development, and MASLD–HCC progression is strongly influenced by metabolic stressors such as obesity, insulin resistance, and lipid overload [38, 39]. Since NMD is regulated by cellular stressors such as ER stress and amino acid deprivation, and many of its targets are involved in stress responses [13, 40, 41], NMD may play a role in CLD and HCC, although it has not yet been described. Our analysis highlights SMG7 as the most dysregulated NMD factor in MASH-HCC, ranking first among VIP-scored genes. Mechanistically, palmitate induced *SMG7* expression, partially mitigated by oleate, consistent with their distinct lipotoxic properties [42–44]. Consistent with these findings, SMG7 expression progressively increased from healthy hepatocytes to HCC cell lines, reaching its highest levels in the most aggressive cellular models, further supporting a role for SMG7 in MASLD-MASH-HCC progression.

SMG7 plays a crucial role in the terminal steps of NMD, acting in concert with UPF1 and other NMD factors to promote mRNA decay in a phosphorylation-dependent manner [13, 45]. While several NMD factors have been described as oncogenes [14, 21, 32, 33, 46, 47], UPF1 has been proposed as a tumor suppressor expressed at lower levels in HCC [17–19, 48, 49]. In our study, however, *UPF1* mRNA expression was unchanged in the internal cohort, but upregulated in TCGA and GSE14520 datasets, with higher protein levels detected in CPTAC.

We found that *SMG7* overexpression promotes HCC aggressiveness. In HCC cell lines, *SMG7* upregulation increased proliferation in Hep3B and SNU-387, and enhanced migration and colony formation, particularly in the more aggressive, mesenchymal-like SNU-387 cells, suggesting a stronger effect in advanced HCC stages. Previous studies indicate that SMG7 modulates stress responses and cell cycle regulation through p53- and ATR–Chk1–related pathways [50–52]. However, in HCC (where p53 is altered in about 30% of cases [53]), SMG7’s role may shift towards tumor promotion, as observed in p53-null Hep3B and mutant p53 SNU-387 cells [54]. Thus, the effects of SMG7 on aggressiveness are highly context dependent. Additionally, in Hep3B cells overexpressing SMG7, reduced UPF1 without changes in its phosphorylation (p-UPF1) or in the expression of NMD targets suggests the maintenance of NMD autoregulation. In SNU-387 cells, high SMG7 levels were associated with a more aggressive phenotype, although this condition did not appear to affect the levels of their canonical NMD target transcripts (*ATF3*, *GADD45A*, and *GADD45B*), suggesting that different pathways may be implicated in the observed changes in cell behavior.

Functionally, *SMG7* silencing reduced proliferation and migration in SNU-387 cells, accompanied by significant upregulation of canonical NMD targets, including *ATF3, GADD45A,* and *GADD45B*, which are key mediators of cellular stress responses, inflammation, and senescence [55–57]. UPF1 was also upregulated in this condition. However, Hep3B cells were largely unaffected and displayed increased UPF1 levels, suggesting a compensatory response.

*UPF1* downregulation in Hep3B and SNU-387 cells produced effects similar to *SMG7* depletion, including compensatory upregulation of SMG7, demonstrating autoregulatory feedback within the NMD machinery, as previously reported [58, 59]. Given the reported low expression of UPF1 in HCC [17, 18, 20, 48, 49] and the compensatory mechanism observed in Hep3B and SNU-387 cells, we evaluated the effects of combined *SMG7* and *UPF1* knockdown in vitro and in vivo. Dual silencing further suppressed aggressiveness compared to single knockdowns in Hep3B and SNU-387 cells. These findings position SMG7 as a central node of NMD whose inhibition sensitizes tumors to UPF1 loss, revealing a combinatorial vulnerability.

Transcriptomic analyses revealed that *SMG7* depletion preferentially activated pathways involved in cytosolic nucleic acid sensing, type I interferon signaling, antigen presentation, and NF-κB activation in SNU-387 cells. Among the most strongly induced genes, *ZBP1* emerged as a candidate mediator linking NMD disruption to inflammatory responses, given its established role in antiviral signaling and inflammatory cell death [60–62]. Consistent with this hypothesis, *ZBP1* clustered within immune-related gene networks, was robustly induced following *SMG7/UPF1* perturbation—particularly upon combined depletion—and showed an inverse correlation with *SMG7* expression across independent HCC cohorts. Although the molecular mechanism underlying this regulation remains to be established, these findings support a functional connection between SMG7 and ZBP1. Consistent with these transcriptomic changes, cross-cohort analyses identified a subset of genes induced by *SMG7* depletion that fulfilled the criteria for putative NMD-regulated transcripts [29] and displayed the opposite expression pattern in human HCC datasets. Although this overlap was partial, it further supports the contribution of SMG7-dependent NMD to transcriptome remodelling in HCC.

Network analyses further highlighted the distinct contribution of *SMG7* within HCC transcriptional programs. Unlike *UPF1*, which was not assigned to any major co-expression module, *SMG7* was identified as a hub gene within a module enriched for biological processes related to metal ion homeostasis and detoxification, cellular stress responses, innate immune signaling, and extracellular matrix and growth regulatory pathways. Moreover, *SMG7* displayed stronger associations with clinically relevant features, including a positive correlation with tumor samples and a negative association with survival time. Furthermore, *ZBP1* emerged as a hub gene in a distinct immune-associated module, supporting the possibility that it represents a downstream effector of SMG7-dependent NMD dysregulation. This functional relationship was further supported by rescue experiments in SNU-387 cells, where *ZBP1* overexpression reduced the increase in proliferation induced by *SMG7* overexpression, indicating that ZBP1 can counteract, at least in part, the pro-tumorigenic effects of SMG7.

Certain limitations of this study warrant consideration. First, while *SMG7* upregulation was consistently identified across early MASLD and MASH stages, the availability of comprehensive, longitudinal human datasets with detailed metabolic characterization remains limited in public repositories. Second, our preclinical xenograft model utilized a small sample size (*n* = 3 mice per group) to minimize animal use in accordance with the 3Rs principles. While this cohort was sufficient to detect statistically significant differences in tumor growth, future studies in larger, immunocompetent models will help to fully explore how this axis modulates the immune microenvironment. Nevertheless, the remarkable consistency of SMG7 upregulation across independent human cohorts underscores its reliability as a hallmark of metabolic hepatocarcinogenesis and a promising therapeutic vulnerability in HCC.

Collectively, our findings identify SMG7 as a key biomarker of chronic liver disease progression and tumor aggressiveness, while combined disruption of SMG7 and UPF1 suppresses tumor growth. Mechanistically, our data support a model in which SMG7 depletion activates antiviral and inflammatory transcriptional programs, potentially through ZBP1 induction, thereby linking NMD impairment to enhanced tumor immunogenicity. Together, these findings highlight NMD modulation as a promising therapeutic strategy to simultaneously restrain tumor aggressiveness and promote antitumor immune responses in HCC.

## Materials and methods

### Patients and samples

A retrospective cohort of 86 patients with HCC who underwent liver transplants (paired tumoral and non-tumoral adjacent tissues, SSPA Biobank, Cordoba node) was analyzed as the discovery cohort, with histological evaluation by two pathologists and clinical data retrieved from medical records (Table S1). Complementary transcriptomic data were obtained from four in silico HCC cohorts: TCGA and three Gene Expression Omnibus (GEO) datasets (GSE6764, GSE14520, and GSE164760). Proteomic data from the UCSC Xena Browser (CPTAC; PDC000199) and SMG7 expression data from two MASLD/MASH GEO cohorts (GSE164760 and GSE135251) were also included. Further methodological details are provided in the Supplementary Material and Methods.

### Cell lines and culture conditions

HCC-derived cell lines Hep3B (HB-8064) and SNU-387 (CRL-2237), as well as the normal-like hepatocyte cell line THLE-2 (CRL-2706), were obtained from the American Type Culture Collection (ATCC, Manassas, Virginia, USA) and cultured as previously reported [8–10, 63]. Further methodological details are provided in the Supplementary Material and Methods.

### Mouse primary hepatocyte isolation

Mouse primary hepatocytes (MPH) were isolated via liver perfusion with collagenase and purified using a Percoll gradient. Cells were then seeded on collagen-coated plates and cultured with treatments for 24 h before analysis as has been reported [64].

### Cell-based model for MASLD progression

MPH were treated for 24 h with 0.5 mM BSA-conjugated palmitate (5 mM, 29,558, Cayman) or BSA alone as a control (5 mM, 29,556, Cayman). Lipid accumulation was assessed by BODIPY 493/503 staining (Cat# 3922, Invitrogen, Eugene, OR). THLE-2 cells were pretreated for 2 h in complete BEBM medium without FBS (0.5% BSA) and then treated for 24 h with 1 mM oleate (O1383-5G, Sigma-Aldrich), palmitate (P9767-5G, Sigma-Aldrich), or an oleate:palmitate mixture (2:1) in complete BEBM without FBS (2.4 mM BSA). Oleate was used to model steatosis (MASLD), the oleate:palmitate mixture to model early MASH, and palmitate to model severe MASH, as previously reported [22–24].

### Transfection and gene silencing

Transient SMG7 and ZBP1 overexpression in HCC cell lines was achieved using a pcDNA3.1 expression vector or empty control vector. Hep3B and SNU-387 cells were transfected using Lipofectamine 2000 or 3000, respectively, and SMG7 or UPF1 silencing was performed using siRNAs and Lipofectamine RNAiMAX. Additional methodological details are provided in the Supplementary Materials and Methods.

### In vitro assays

Proliferation, migration, colony formation, and tumorsphere assays were performed in Hep3B and SNU-387 cells under standardized conditions to evaluate growth, motility, clonogenic capacity, and self-renewal, with results quantified using fluorescence-based, wound healing, crystal violet staining, and tumorsphere imaging analyses, respectively, as previously described [8–10, 63]. Additional information can be found in the Supplementary Material and Methods.

### RNA isolation and sequencing, qPCR, and western blotting

Total RNA was isolated from cell lines, primary hepatocytes, xenografts, and FFPE samples for cDNA synthesis and gene expression analysis by standard or high-throughput qPCR (using the genes listed in Table S9) and normalized using GeNorm-selected housekeeping genes (β-actin and Hypoxanthine Phosphoribosyltransferase 1). RNA from SMG7-silenced and control SNU-387 cells was extracted, quality-controlled, and subjected to paired-end RNA sequencing, followed by read quantification and DESeq2 normalization. Protein expression of SMG7, UPF1, phospho-UPF1, and ZBP1 in transfected HCC cell lines was analyzed by Western blot and normalized to β-tubulin. Additional methodological details are provided in the Supplementary Materials and Methods.

### Xenograft model

Twenty-seven-week-old male nude mice were subcutaneously injected with 5 × 10⁶ Hep3B cells, and once tumors reached ~100 mm^3^, mice were treated with siRNAs targeting SMG7, UPF1, both, or control siRNA using AteloGene. Tumor growth and body weight were monitored until tumors reached ~1000 mm^3^, after which mice were euthanized and tumors were collected for analysis. For additional details, see the Supplementary Materials and Methods.

### Bioinformatic and statistical analysis

Exploratory analyses were conducted with MetaboAnalyst 5.0 using PLS-DA (Partial Least Squares Discriminant Analysis) and VIP (Variable Importance in Projection) scores to indicate the relative contribution of each variable to group discrimination in the PLS-DA model. Pathway enrichment was assessed via GSEA (v4.3.2), PPI networks were generated with STRING, and weighted gene co-expression network analysis (WGCNA) was performed on variance-stabilized TCGA-LIHC RNA-seq data after filtering low-expression and low-variance genes and removing outlier samples, using an unsigned network (β = 6) to identify co-expression modules and assess their associations with clinical traits. Categorical clinical traits were numerically encoded prior to correlation analysis, and days to death was included as a continuous variable. Co-expression networks were exported from WGCNA to Cytoscape for visualization, and functional enrichment analysis of each module was performed using g:Profiler to identify the main biological pathways associated with each co-expression module.

Data are presented as mean ± SEM. Statistical analyses were conducted using GraphPad Prism v10.3.1. Normality was assessed using the Shapiro–Wilk test and comparisons between two groups were performed using unpaired Student’s t-test or Mann–Whitney U test, as appropriate. Tumor growth curves were analyzed by two-way ANOVA (treatment × time) with Fisher's LSD post hoc test for pairwise comparisons at each time point. Correlations were evaluated using Pearson’s or Spearman’s coefficients according to data distribution. Receiver operating characteristic (ROC) curve analyses were performed to assess diagnostic performance, and area under the curve (AUC) values were calculated. All tests were two-sided, and a *P* value < 0.05 was considered statistically significant. For transcriptomic analyses, adjusted *P* values (Benjamini–Hochberg false discovery rate, FDR < 0.05) were used where indicated. Detailed methods are provided in the Supplementary Materials and Methods.

## Supplementary Information

Supplementary Material 1.

Supplementary Material 2.

## Data Availability

The raw RNA-seq count matrix generated in this study is publicly available as Supplementary Material 1 in Figshare at https://doi.org/10.6084/m9.figshare.30657863, enabling independent differential expression and downstream analyses. The publicly available transcriptomic datasets analyzed in this study were obtained from the NCBI Gene Expression Omnibus (GEO) under accession numbers GSE6764, GSE14520, GSE135251, and GSE164760, and from the TCGA Liver Hepatocellular Carcinoma project (TCGA-LIHC). Proteomic data were obtained from the NCI Clinical Proteomic Tumor Analysis Consortium/Proteomic Data Commons under study identifier PDC000199. De-identified individual-level clinical data from the discovery cohort are not publicly available because of ethical and privacy restrictions but may be available from the corresponding author upon reasonable request and subject to approval by the relevant ethics committee and biobank. Detailed descriptions of the data sources and characteristics of the cohorts are provided in the “Patients and samples” section of the Supplementary Material and Methods. All other data supporting the findings of this study are available within the article and its Supplementary Information.

## Abbreviations

AUC: Area under the curve
DECID: Decay-inducing complex
EJC: Exon junction complex
HCC: Hepatocellular carcinoma
HBV: Hepatitis B virus
HCV: Hepatitis C virus
KD: Knockdown
MASH: Metabolic dysfunction-associated steatohepatitis
MASLD: Metabolic dysfunction-associated steatotic liver disease
MPH: Mouse primary hepatocytes
NMD: Nonsense-mediated decay
NTAT: Non-tumoral adjacent tissue
OA: Oleic acid
PA: Palmitic acid
PCA: Principal component analysis
PPI: Protein–protein interaction
PTC: Premature termination codon
RNA-seq: RNA sequencing
ROC: Receiver operating characteristic
SCR: Scramble
SMG7: Suppressor with Morphogenetic Effect on Genitalia 7
SURF: SMG1–UPF1–eRF1–eRF3 complex
UTR: Untranslated region
VIP: Variable importance in projection
ZBP1: Z-DNA-binding protein 1
