# Supplementary material for: Suppressor with morphogenetic effect on genitalia 7 (SMG7) drives hepatocellular carcinoma progression through context-dependent nonsense-mediated decay (NMD) dysregulation
Source: Mol Biomed. 2026 Oct 12;7:206. doi: 10.1186/s43556-026-00577-3 (PMC13658749; doi:10.1186/s43556-026-00577-3)
Supplement: Supplementary file 1 [file 43556_2026_577_MOESM2_ESM.docx]

**Title: Suppressor with morphogenetic effect on genitalia 7 (SMG7) drives hepatocellular carcinoma progression through context-dependent nonsense-mediated decay (NMD) dysregulation**

**Authors**: Betsaida Ojeda-Pérez^1,2,3,4^, Víctor J. Fernández-Ramírez^1,2,3^, María I. Pozo-Relaño^1,2,3,4^, Belén Gracia-Herencia^2,3,4^, Izabela Hawro^5^, Jose Córdoba-Chacón^5^, Raul M. Luque^1,2,3,4^, Manuel L. Rodriguez-Peralvarez^2,6,7^, Juan L. López-Cánovas^1,2,3,4,*^, Manuel D. Gahete^1,2,3,4,*^.

**Affiliations:** ^1^Department of Cell Biology, Physiology and Immunology, University of Cordoba, Cordoba, Spain; ^2^Maimonides Institute of Biomedical Research of Cordoba (IMIBIC), Cordoba, Spain; ^3^Reina Sofia University Hospital (HURS), Cordoba, Spain; ^4^CIBER Physiopathology of Obesity and Nutrition (CIBERobn), Madrid, Spain; ^5^Department of Medicine, University of Illinois at Chicago, Chicago, IL, USA; ^6^Department of Hepatology and Liver Transplantation, Reina Sofía University Hospital, Córdoba, Spain; ^7^CIBER Hepatic and Digestive Diseases (CIBERehd), Madrid, Spain.

* These authors jointly supervised this work and serve as co-corresponding authors.

**Corresponding Authors:** Manuel D. Gahete (Email Address: [bc2gaorm@uco.es](mailto:bc2gaorm@uco.es)) or Juan L. López-Cánovas (Email Address: b32locaj@uco.es).

**Journal name:** Molecular Biomedicine

**Supplementary Material and Methods**

**Patients and samples**

A retrospective cohort comprising 86 patients with HCC was used as the discovery cohort. Formalin-fixed, paraffin-embedded (FFPE) samples of both tumor tissue and non-tumor adjacent tissue (NTAT) were obtained from liver transplant resections. Samples were obtained from the Andalusian Biobank (Cordoba Node) and histologically evaluated by two pathologists. Clinical data were retrieved from electronic medical records (Table S1). This retrospective cohort study was approved by the institutional ethics committee (protocol numbers PI20/01301 and PI23/00652), and written informed consent was obtained.

mRNA expression data for NMD factors were obtained from four *in silico* HCC cohorts with different etiologies: Liver Hepatocellular Carcinoma samples (LIHC) from The Cancer Genome Atlas (TCGA) with 371 primary tumor and 50 solid tissue normal [1], and the Gene Expression Omnibus (GEO) database: GSE6764 (10 normal liver vs. 35 HCC) [2], GSE14520 (220 normal liver vs. 225 HCC) [3], and GSE164760 (53 MASH-HCCs vs. 29 adjacent non-tumor MASH liver samples and 74 MASH samples vs. 6 healthy livers as a control) [4]. Finally, GSE135251 (liver biopsies: 206 NAFLD cases and 10 controls) was used only for SMG7 expression [5]. Proteomic data from paired tumor and adjacent liver tissues of 159 patients with HBV-related HCC were retrieved from the Cancer Institute’s Clinical Proteomic Tumor Analysis Consortium (CPTAC) portal (PDC000199) [6].

**RNA isolation, qPCR dynamic array, standard qPCR and sequencing**

Total RNA from the discovery cohort (FFPE tissue samples) was isolated using the Maxwell FFPE Purification Kit (Thermo Fisher). High-throughput gene expression analysis (96 genes × 96 samples) was performed using a microfluidic-based qPCR dynamic array platform (Fluidigm, San Francisco, CA, USA). Preamplification, exonuclease treatment, and qPCR using the dynamic array were carried out as previously reported [7–9]. To control for reverse transcription efficiency, expression levels of three housekeeping genes were analyzed with GeNorm 3.3 to derive a normalization factor.

RNA from cell lines was extracted with Trizol reagent (Thermo Fisher), and for xenograft tumors with the AllPrep DNA/RNA/Protein Kit (QIAGEN), following the manufacturer’s protocols. DNase treatment (Promega, Barcelona, Spain) was performed before reverse transcription. RNA concentration and purity were measured with a NanoDrop 2000 spectrophotometer (Thermo Fisher), and complementary DNA (cDNA) was synthesized using the ReverAid First-Strand cDNA Synthesis Kit (Thermo Fisher) at 50 ng/μl. Standard qPCR was conducted according to previously described protocols [10] to assess mRNA expression in cell lines, mouse primary hepatocytes, and xenograft tumor models. Amplification was carried out with the iTaq Universal SYBR Green Supermix (#1725124, Bio-Rad) and the CFX Duet Real-Time PCR system. Gene copy numbers were quantified and normalized using a normalization factor derived from GeNorm 3.3, based on the expression levels of two housekeeping genes, β-actin and hypoxanthine phosphoribosyltransferase 1 (HPRT1). Custom primers for human and mouse transcripts were designed using Primer3 (Applied Biosystems, Foster City, CA, USA) (see Table S9). All primers detect total transcript levels, including all isoforms*. ATF3*, *GADD45A*, and *GADD45B* are known NMD target genes whose transcripts are directly degraded by the NMD pathway. A primer pair targeting the unspliced pre-mRNA of *ZBP1* was used to quantify nascent transcription independently of mature mRNA levels.

For RNA-sequencing (RNA-seq), total RNA was isolated from SMG7-silenced SNU-387 cells (n = 3) and scramble-transfected controls (n = 3) using the AllPrep DNA/RNA/Protein Kit (QIAGEN). RNA quality and quantity were initially measured using NanoDrop 2000 spectrophotometer (Thermo Fisher), and integrity was verified with the Agilent 2200 TapeStation System (Agilent Technologies). Only samples with an RNA Integrity Number (RIN) ≥ 7.0 were retained for downstream transcriptomic analyses. RNA-seq was performed by Novogene (Cambridge, UK). Total RNA was subjected to poly(A) selection to enrich mRNA, followed by enzymatic fragmentation. First- and second-strand cDNA synthesis was performed according to standard protocols. Subsequent steps, including end-repair, A-tailing, and adaptor ligation, were conducted as part of the library construction workflow. Amplified libraries were purified and quality-checked prior to sequencing. Paired-end sequencing (2 × 150 bp) was performed on the Illumina NovaSeq X Plus platform. Raw data were provided in FASTQ format through secure cloud-based transfer for downstream analysis (described below).

**Western blot analysis**

THLE-2 and HCC cell lines were seeded in 6-well plates (300,000–400,000 cells/well) and processed for Western blot analysis 24 hours after seeding or transfection with SMG7 plasmid or siRNAs targeting *SMG7* or *UPF1*. Proteins were extracted using pre-warmed SDS-DTT buffer (62.5 mM Tris-HCl, 2% SDS, 20% glycerol, 100 mM DTT, and 0.005% bromophenol blue). Lysates were sonicated for 10 seconds and boiled for 5 minutes at 95 °C. Proteins were then separated by SDS-PAGE and transferred to nitrocellulose membranes (Millipore, Billerica, MA, USA). Membranes were blocked with 5% non-fat dry milk in Tris-buffered saline with 0.05% Tween-20 and incubated overnight at 4 °C with the following primary antibodies: SMG7 (#PA5-101223, Invitrogen), UPF1 (#12040S, Cell Signalling), phospho-UPF1 (Ser1107, #84283S, Cell Signalling), ZBP1 (#13285-1-AP, Proteintech), and beta-tubulin (#2128S, Cell Signalling). Appropriate HRP-conjugated secondary antibodies (goat anti-rabbit IgG, #7074S, Cell Signalling) were used. Protein bands were visualized using a chemiluminescence analysis system (SCG-W5000, Servicebio) with molecular weight markers (Precision Plus Protein Dual Color Standards, #1610374, Bio-Rad) as molecular-weight references. Densitometric analysis was performed using ImageJ (FIJI). Band intensities were normalized to β-tubulin expression. All experiments were conducted in at least three independent biological replicates.

**Mouse primary hepatocyte isolation**

Mouse primary hepatocytes (MPH) were isolated using an *in situ* liver perfusion system (Harvard Apparatus) followed by collagenase digestion. Chow-fed 5-6-month-old male mice were anesthetized via intraperitoneal injection of ketamine and xylazine (100/10 mg/kg) and euthanized via exsanguination during liver perfusion, which facilitated the isolation of MPH, as previously reported [11]. The liver was then perfused through the inferior vena cava with a perfusion buffer consisting of calcium- and magnesium-free Hanks' Balanced Salt Solution (HBSS) without phenol red, supplemented with 0.5 mM EDTA (137 mM NaCl, 5.33 mM KCl, 0.44 mM KH2PO4, 0.33 mM Na2HPO4, 0.5 mM ethylenediaminetetraacetic acid, pH 7.3). This was followed by perfusion with a digestion buffer containing collagenase (25 µg/ml collagenase type I and II Liberase^TM^, 05401119001, Sigma Aldrich) prepared in Dulbecco’s Modified Eagle Medium (DMEM) supplemented with 1× penicillin/streptomycin and 15 mM HEPES.

Following digestion, the liver was excised, mechanically dissociated, filtered, and centrifuged to collect hepatocytes in complete DMEM (10% fetal bovine serum, L-glutamine (2 mM), and 1× penicillin/streptomycin). To enhance cell viability and purity, a Percoll gradient purification step was performed. All procedures were carried out under sterile and cold conditions to preserve cell viability. Isolated hepatocytes were counted and seeded (2 × 10^5^ cells/well) in collagen-coated 12-wells culture plates prepared with 50 μg/ml rat-tail type I collagen (354236, Corning) in 0.02 M of glacial acetic acid. After a 4-hour incubation at 37 °C in a humidified CO₂ incubator, the isolation medium (complete DMEM) was replaced with complete DMEM/F-12 supplemented with 10% fetal bovine serum (FBS), 1× penicillin/streptomycin, and 1% L-glutamine (2 mM) containing the desired treatment, and cells were incubated for an additional 24 hours to follow up with the next procedures. Mice were housed in groups at UIC’s Biological Resources Facility under controlled environmental conditions, including a stable temperature of 22–24°C, regulated humidity, and a 14-hour light/10-hour dark cycle.

Mouse primary hepatocyte isolation procedures were approved by the Animal Care Committee (ACC) of the University of Illinois Chicago (UIC; ACC protocol no. 22-189) and were conducted in accordance with the National Institutes of Health Guide for the Care and Use of Laboratory Animals.

**Cell lines and culture conditions**

Hep3B cells (HB-8064, lot no. 63194732) were cultured in Minimum Essential Medium (MEM) (Gibco™, Thermo Fisher Scientific, Madrid, Spain), supplemented with 10% fetal bovine serum (FBS, Sigma-Aldrich, Madrid, Spain) and 1% antibiotic-antimycotic solution (A5955, Sigma-Aldrich) plus 1% sodium pyruvate (Gibco™, Thermo Fisher Scientific). SNU-387 cells (CRL-2237, lot no. 62596267) were cultured in complete Roswell Park Memorial Institute medium (RPMI 1640) (15-040-CV, Corning) also supplemented with 10% FBS, 1% antibiotic-antimycotic and 1% L-glutamine (Sigma-Aldrich).

The THLE-2 cell line (CRL-2706, lot no. 70026207), originating from primary normal liver cells infected with SV40 large T antigen, was cultured in Bronchial Epithelial Cell Basal Medium (BEBM) supplemented with components from the BEGM BulletKit (Lonza/Clonetics, USA; CC-3170) and was maintained as described by ATCC. All cells were maintained at 37°C in a 5% CO₂ atmosphere and routinely tested for mycoplasma contamination.

**Transfection and gene silencing**

Transient overexpression of *SMG7* or *ZBP1* was achieved by transfecting cells with 1 μg of a pcDNA3.1 expression vector encoding human *SMG7* (NM_201568.3) or *ZBP1* (NM_030776.3) (GenScript, Oxford, UK) or an empty pcDNA3.1 vector as a mock control in HCC cell lines. For transfection, 350,000 Hep3B cells were seeded in 6-well plates and transfected using Lipofectamine™ 2000 reagent (Thermo Fisher, 11668019), whereas 400,000 SNU-387 cells were seeded per well and transfected using Lipofectamine™ 3000 (Invitrogen, L3000015).

For gene silencing, custom-designed siRNAs (25 nM; Horizon, Dharmacon) targeting *SMG7* (5′‑CGAUUUGGAAUACGCUUUA-3′) and *UPF1* (5′-GAUGCAGUUCCGCUCCAUU-3′) and a nontargeting siRNA control (ON-TARGET plus Non-targeting Pool, D-001810-10-20, Horizon, Dharmacon) were used, based on sequences described by Boehm et al. (2021) [12] and Kurosaki et al. (2014) [13], respectively. Transfections were performed using Lipofectamine RNAiMAX (Thermo Fisher) in 250,000 Hep3B cells or 300,000 SNU-387 cells.

**Cell proliferation**

Cell proliferation was assessed using a resazurin-based fluorometric assay (#CA035; Canvax Biotech, Córdoba, Spain). At 24 hours post-transfection, 3,000 cells/well of SNU-387 or Hep3B were seeded in 96-well plates with 10% FBS medium. After overnight incubation, the medium was replaced with serum-free medium for 10–12 hours to synchronize the cell cycle. Resazurin (10%) was added and incubated for 3 hours before measuring baseline fluorescence. Cells were then cultured in 5% FBS, and fluorescence was measured at 24, 48, and 72 hours. Each experiment included at least three technical and biological replicates.

**Cell migration**

Cell migration was measured using a wound healing assay in Incucyte® 96-well plates (Sartorius). Post-transfection, cells were seeded to reach 90% confluence within 24 hours. After 2 hours in serum-free medium, wounds (~700–800 μm) were made, and images were taken at 0 and 24 hours. The wound area recovery was then evaluated using the ImageJ (FIJI). Each experiment included at least three technical and biological replicates.

**Colony formation**

At 24 hours post-transfection, cells were seeded at low density (1000 cells/well) in 6-well plates and cultured for up to 10 days in complete medium, refreshed every 2–3 days. Colonies were fixed and stained with a solution of crystal violet (0.5%) and glutaraldehyde (6%), then the number was quantified using ImageJ (FIJI). Each experiment included two technical and at least three biological replicates.

**Tumorsphere formation**

Hep3B cells (5,000 cells/well) or SNU-387 cells (10,000 cells/well) were seeded in ultra-low attachment 24-well plates (Sigma-Aldrich) and cultured in 10% FBS medium supplemented with EGF (E9644-.2MG, Sigma-Aldrich) (Hep3B) and B27 (12587-010, Thermo Fisher) (Hep3B and SNU-387). Medium was refreshed every 3 days. After 10 days, 4× images were taken and tumorsphere number and size were analyzed with ImageJ (FIJI). Each condition included two technical and at least three biological replicates.

**Xenograft model**

Twenty-seven-week immunodeficient nude mice (Foxn1^nu^/Foxn1^nu^, Janvier Labs, Le Genest-Saint-Isle, France), housed under standard specific pathogen-free (SPF) conditions with free access to food and water, were subcutaneously injected in both flanks with 5 × 10⁶ Hep3B cells per site. The cells were suspended in a 1:1 mixture of culture medium and basement membrane extract (BME, Trevigen, Gaithersburg, MD, USA). Once tumors reached an approximate volume of 100 mm³ (at day 16), mice were randomized (n = 3 per group) and received peritumoral subcutaneous injections of siRNAs targeting *SMG7*, *UPF1*, or their combination. A non-targeting pool of siRNAs served as negative (scramble) control (SCR, n = 3). All transfections were performed using the AteloGene *in vivo* transfection kit (KKN1494, REPROCELL). Tumor volume and body weight were monitored every other day. When tumors reached an approximate volume of 1000 mm³, mice were euthanized. Tumors were excised, and representative tissue sections were snap-frozen for further analysis. The animal procedures were conducted in accordance with European Regulations for Animal Care under the approval of the University Research Ethics Committee (protocol no. 08/03/2022/033).

**Bioinformatic and statistical analyses**

Initial exploratory analyses were conducted with MetaboAnalyst 5.0. Partial Least Squares Discriminant Analysis (PLS-DA), a supervised dimensionality reduction technique, was employed to identify variables that best distinguish between experimental groups (e.g., NTAT vs. tumor). This method enhances separation by maximizing variance through linear regression modelling. The Variable Importance in Projection (VIP) score was calculated to estimate the contribution of each variable to the PLS-DA model, based on the weighted sum of squared correlations between variables and the model components.

After RNA sequencing, raw sequencing reads were quality-checked using FastQC (v0.12.1) [14]. Adapter sequences and low-quality bases were trimmed with TrimGalore (v0.6.10) [15] under stringent filtering conditions. Transcript quantification was performed with Salmon (v1.10.2) [16] in quasi-mapping mode against the Ensembl GRCh38 transcriptome, providing accurate and bias-corrected expression estimates in transcripts per million (TPM). Transcript-level TPMs were summarized to gene-level counts using the tximport package (v1.34.0) from Bioconductor [17]. Genes with low expression (mean log₂ counts per million < 0) were excluded from downstream analyses. Gene counts were normalized using the internal size factor estimation method implemented in DESeq2 (v1.46.0), which adjusts for differences in library size across samples [18]. Differential expression analysis was subsequently performed, and differentially expressed genes (DEGs) were defined as those exhibiting an adjusted P-value < 0.05 and an absolute fold change > 1.50. Multiple testing correction was applied using the Benjamini-Hochberg procedure to control the false discovery rate. The raw counts dataset is available in Supplementary Material 4.

Pathway enrichment was assessed with Gene Set Enrichment Analysis (GSEA) (version 4.3.2) on RNA-seq data, comparing SMG7 knockdown (SMG7^KD^) versus controls (scramble), as implemented in the GenePattern platform [19,20]. Enrichment scores were evaluated against null distributions derived from phenotype-based permutations, and significance thresholds were adjusted to account for multiple comparisons. Analyses were performed in R (v4.4.2) [21], and full code is available upon request.

Protein-protein interaction (PPI) networks were generated using the Search Tool for the Retrieval of Interacting Genes/Proteins (STRING; https://www.string-db.org/), focusing on genes that were significantly upregulated in *SMG7*-silenced SNU-387 cells. STRING clustering was used to group genes by shared molecular functions.

Data are presented as mean ± standard error of the mean (SEM), expressed either as fold-change (log₂) or as values normalized to controls (set to 100%). Normality was assessed using the Shapiro-Wilk test for small samples groups or D'Agostino-Pearson test for larger sample groups, and depending on the distribution, either parametric (Student’s t-test) or non-parametric (Mann–Whitney U test) methods were applied. Bivariate correlations between quantitative variables were evaluated using Spearman’s or Pearson’s coefficients based on data normality. A two-tailed P value < 0.05 was considered statistically significant. For RNA-seq differential expression and enrichment analyses, Benjamini–Hochberg-adjusted P values (FDR < 0.05) were used. Longitudinal tumor growth data were analyzed by two-way ANOVA using treatment group and time as independent variables. When appropriate, Fisher's least significant difference (LSD) post hoc test was performed to compare treatment groups at individual time points. Additional statistical analyses were performed with GraphPad Prism v10.3.1 (La Jolla, CA, USA), including univariate and multivariate ROC curve analyses for biomarker evaluation.

**Weighted gene co-expression network analysis**

Raw RNA-seq count data from TCGA-LIHC were imported and subjected to a low-expression filtering step in which genes with fewer than 10 counts in at least 20% of samples were excluded prior to normalization. Library-size normalization and variance stabilization were performed using DESeq2 (v1.46.0), applying the variance-stabilizing transformation (VST) with blind dispersion estimation disabled. Genes were subsequently filtered to retain those exceeding the 95th percentile of row-wise variance across the VST-normalized matrix. Sample quality was assessed by unsupervised hierarchical clustering of Euclidean distances using average linkage; samples above a cut height of 135 were removed as outliers, yielding a final cohort of 419 samples. Weighted gene co-expression network analysis was performed using the WGCNA R package (v1.74) [22]. Soft-thresholding power was selected by evaluating candidate powers (1–20) using the scale-free topology criterion and choosing the lowest value at which the scale-free topology fit (R²) approached 0.90; β = 6 was selected. An unsigned co-expression network was constructed using blockwiseModules with the following parameters: maximum block size of 4,000 genes, minimum module size of 30 genes, deepSplit = 2, pamRespectsDendro = FALSE, reassignThreshold = 0, and mergeCutHeight = 0.25. Module eigengenes were computed using moduleEigengenes and ordered by eigengene dissimilarity. Module–trait associations were quantified by Pearson correlation between module eigengenes and numerically encoded clinical variables, with significance assessed via Student's t-distribution approximation. Gene module membership (kME) was computed as the Pearson correlation between each gene's expression profile and each module eigengene. Clinical traits were numerically encoded to allow for correlation analysis, including vital status (1: alive, 2: dead), sample type (1: non-tumoral, 2: tumoral), gender (1: female, 2: male), and race (1: American Indian/Alaska Native, 2: Asian, 3: Black/African American, 4: not reported, 5: unknown, 6: White). The fibrosis score was modelled ordinally from no fibrosis (1) to established cirrhosis (5), and days to death was included as a continuous variable.

Co-expression networks for each WGCNA module were exported using the exportNetworkToCytoscape function from the WGCNA R package [22], filtering gene pairs by a topological overlap (TOM) threshold of 0.1, and the resulting edge list, with module color embedded as a node attribute, was imported into Cytoscape (v3.10.x; [23]) for network visualization using a force-directed layout. Functional enrichment analysis for each module was performed directly within Cytoscape using the built-in Tools → Enrichment Table → Gene Enrichment function, which queries the g:Profiler database [24] against Gene Ontology, Reactome, WikiPathways, and KEGG (for the turquoise module, which returned no results in Cytoscape, the same gene list was submitted to the g:Profiler web interface using identical settings); enriched pathways were ranked by intersection size and adjusted p-value, and the most representative terms for each module were compiled to summarize module-level biological function.

**Supplementary Tables**

**Table S1.** Demographic and clinical profile of patients with hepatocellular carcinoma (HCC) enrolled in the discovery cohort (*n* = 86). Data are presented as number and percentage of patients, unless otherwise indicated. IQR, interquartile range; HCV, hepatitis C virus; HBV, hepatitis B virus.

| **Characteristic** | **n (%)** |
| --- | --- |
| Patients | 86 |
| **Etiology** |  |
| HCV | 30 (36.1) |
| Alcohol | 21 (25.3) |
| HBV | 11 (13.3) |
| Other | 5 (6.0) |
| HCV + Alcohol | 9 (10.8) |
| HBV + Alcohol | 1 (1.2) |
| HCV + Other | 3 (3.6) |
| Alcohol + Other | 0 (–) |
| **Histological differentiation** |  |
| Well differentiated | 30 (35.3) |
| Moderately differentiated | 50 (58.8) |
| Poorly differentiated | 5 (5.9) |
| Portal hypertension | 44 (51.2) |
| Microvascular invasion | 33 (39.8) |
| Treated before surgery | 23 (26.4) |
| Recurrence | 39 (47.0) |
| Death | 50 (61.0) |

**Table S2.** Putative NMD-regulated transcripts identified by Boehm et al. [25] that are upregulated following *SMG7* depletion in SNU-387 cells, and their log_2_ fold change (logFC) values across four HCC transcriptomic cohorts. Genes are listed alphabetically. AS-NMD, alternative splicing-coupled NMD.

| **Gene** | **NMD mechanism** | **TCGA logFC** | **GSE14520 logFC** | **GSE164760 logFC** | **GSE6764 logFC** | **Sig. cohorts*** |
| --- | --- | --- | --- | --- | --- | --- |
| *DDX60L* | Novel isoform (mixed) | -0.813 | N/F | -0.171 | 0.545 | 1 |
| *DHX58* | Predicted NMD | -0.737 | -0.388 | -0.207 | 0.061 | 2 |
| *ETV7* | Novel isoform (NMD) | -0.064 | -0.108 | -0.048 | 0.623 | 1 |
| *GBP3* | AS-NMD | -0.304 | N/F | -0.021 | 0.393 | 0 |
| *HELZ2* | AS-NMD | N/F | N/F | N/F | 0.166 | 0 |
| *HERC6* | Predicted NMD | -0.872 | -0.209 | 0.168 | 1.624 | 3 |
| *IFI44* | AS-NMD | -0.590 | -0.576 | -0.055 | 1.940 | 3 |
| *IFIH1* | AS-NMD | -0.408 | -0.089 | -0.020 | 0.768 | 1 |
| *LAP3* | AS-NMD (mixed) | -1.119 | -0.520 | 0.034 | 0.163 | 2 |
| *PARP12* | AS-NMD | 0.353 | 0.881 | 0.331 | 1.507 | 4 |
| *PLSCR1* | AS-NMD (mixed) | -0.991 | -0.285 | -0.020 | -0.066 | 2 |
| *PNPT1* | AS-NMD | 0.150 | N/F | 0.040 | 0.496 | 1 |
| *PPM1K* | AS-NMD | -1.799 | N/F | -0.088 | -1.177 | 2 |
| *SHFL* | AS-NMD | -0.753 | -0.986 | 0.062 | -0.407 | 2 |
| *SMDT1* | AS-NMD (mixed) | -0.139 | N/F | -0.048 | -0.796 | 1 |
| *STAT1* | AS-NMD (mixed) | 0.018 | 0.535 | 0.100 | 1.407 | 2 |
| *TRIM25* | AS-NMD | 0.908 | -0.201 | 0.019 | 0.312 | 2 |

* Number of cohorts in which the gene reached statistical significance (adj.P.Val < 0.05). adj.P.Val, adjusted P value (Benjamini–Hochberg correction); logFC, log2 fold change; N/F, not detected in cohort.

**Table S3.** Number of downregulated genes identified among genes upregulated following *SMG7* depletion in SNU-387 cells, stratified by log_2_ fold change (logFC) threshold and the minimum number of cohorts in which statistical significance was reached.

| **logFC threshold** | **≥1 cohort** | **≥2 cohorts** | **≥3 cohorts** | **4 cohorts** |
| --- | --- | --- | --- | --- |
| Any down (logFC < 0, adj.P < 0.05) | 62 | 36 | 3 | 0 |
| logFC < −0.58 | 45 | 12 | 1 | 0 |
| logFC < −1.0 | 13 | 4 | 0 | 0 |
| logFC < −2.0 | 2 | 1 | 0 | 0 |

* Statistical significance was defined as adj.P.Val < 0.05 (Benjamini–Hochberg method). A logFC of ±0.58 corresponds to an approximately 1.5-fold change in expression. Each cell shows the number of genes meeting the indicated logFC threshold and significance criterion in ≥ N cohorts. Three genes were not detected in any cohort (PLA2G4B, SPDYE2B, TENT5A) and are excluded from all counts.

**Table S4.** Number of upregulated genes identified among genes upregulated following *SMG7* depletion in SNU-387 cells, stratified by log_2_ fold change (logFC) threshold and the minimum number of cohorts in which statistical significance was reached.

| **logFC threshold** | **≥1 cohort** | **≥2 cohorts** | **≥3 cohorts** | **4 cohorts** |
| --- | --- | --- | --- | --- |
| Any up (logFC > 0, adj.P < 0.05) | 27 | 8 | 3 | 1 |
| logFC > 0.58 | 23 | 5 | 0 | 0 |
| logFC > 1.0 | 17 | 1 | 0 | 0 |
| logFC > 2.0 | 6 | 0 | 0 | 0 |

* Statistical significance was defined as adj.P.Val < 0.05 (Benjamini–Hochberg method). A logFC of ±0.58 corresponds to an approximately 1.5-fold change in expression. Each cell shows the number of genes meeting the indicated logFC threshold and significance criterion in ≥ N cohorts. Three genes were not detected in any cohort (PLA2G4B, SPDYE2B, TENT5A) and are excluded from all counts.

**Table S5.** Interferon-related and immune genes significantly dysregulated in ≥ 3 of 4 HCC transcriptomic cohorts among genes upregulated following *SMG7* depletion in SNU-387 cells (*n* = 10). logFC, log_2_ fold change.

| **Gene** | **TCGA logFC** | **GSE14520 logFC** | **GSE164760 logFC** | **GSE6764 logFC** | **Sig. cohorts*** |
| --- | --- | --- | --- | --- | --- |
| *PARP12* | 0.35 | 0.88 | 0.33 | 1.51 | 4 |
| *LY6E* | -3.20 | -2.08 | -0.37 | -1.09 | 3 |
| *MAP2* | 1.04 | 0.18 | 0.68 | 2.44 | 3 |
| *IFI44L* | -1.13 | -0.52 | -0.28 | 2.16 | 3 |
| *IFI44* | -0.59 | -0.58 | -0.06 | 1.94 | 3 |
| *OAS2* | -0.82 | -0.33 | -0.06 | 1.89 | 3 |
| *HERC6* | -0.87 | -0.21 | 0.17 | 1.62 | 3 |
| *TAP1* | 0.76 | 0.44 | -0.15 | 1.45 | 3 |
| *AKAP9* | -0.79 | -0.69 | 0.13 | -0.89 | 3 |
| *CX3CL1* | -0.71 | -0.14 | -0.34 | 0.24 | 3 |

* Number of cohorts in which the gene reached statistical significance (adj.P.Val < 0.05). adj.P.Val, adjusted P value (Benjamini–Hochberg correction); logFC, log_2_ fold change; N/F, not detected in the cohort.

**Table S6.** Interferon-related and immune genes significantly dysregulated in exactly 2 of 4 HCC transcriptomic cohorts among genes upregulated following *SMG7* depletion in SNU-387 cells (*n* = 39). logFC, log_2_ fold change.

| **Gene** | **TCGA logFC** | **GSE14520 logFC** | **GSE164760 logFC** | **GSE6764 logFC** | **Sig. cohorts*** |
| --- | --- | --- | --- | --- | --- |
| *TRIM22* | -1.25 | -1.70 | -0.37 | 1.11 | 2 |
| *EPSTI1* | -1.01 | N/F | 0.01 | 2.04 | 2 |
| *PPM1K* | -1.80 | N/F | -0.09 | -1.18 | 2 |
| *IFIT1* | -1.08 | -0.88 | 0.01 | 1.74 | 2 |
| *HERC5* | -1.70 | -0.75 | 0.03 | 0.81 | 2 |
| *IFITM1* | -1.26 | -1.28 | -0.29 | 0.33 | 2 |
| *XAF1* | -0.71 | -1.00 | 0.21 | 0.94 | 2 |
| *CD69* | -1.73 | -0.55 | -0.22 | 0.20 | 2 |
| *OAS1* | -0.80 | -0.57 | -0.12 | 1.17 | 2 |
| *RNF213* | 0.88 | N/F | 0.21 | 0.89 | 2 |
| *OASL* | -0.70 | -0.59 | -0.02 | 1.32 | 2 |
| *OAS3* | -0.02 | 0.25 | 0.66 | 1.59 | 2 |
| *TLR3* | -1.51 | -0.34 | 0.00 | -0.60 | 2 |
| *BST2* | -0.72 | -0.88 | -0.01 | 0.80 | 2 |
| *TAP2* | 0.59 | 0.07 | 0.35 | 1.27 | 2 |
| *APOL6* | -1.30 | -0.68 | 0.07 | -0.23 | 2 |
| *IFIT2* | -0.53 | -0.32 | 0.00 | 1.41 | 2 |
| *SHFL* | -0.75 | -0.99 | 0.06 | -0.41 | 2 |
| *DDX60* | -0.63 | -0.13 | 0.04 | 1.31 | 2 |
| *STAT1* | 0.02 | 0.53 | 0.10 | 1.41 | 2 |
| *IFI16* | -0.60 | -0.34 | -0.21 | 0.85 | 2 |
| *IFIT3* | -0.38 | -0.07 | -0.07 | 1.45 | 2 |
| *IRF7* | -0.58 | -0.61 | -0.22 | 0.54 | 2 |
| *LAP3* | -1.12 | -0.52 | 0.03 | 0.16 | 2 |
| *DTX3L* | -0.18 | N/F | 0.74 | 0.41 | 2 |
| *CEACAM1* | -0.59 | -0.37 | 0.14 | 0.62 | 2 |
| *UBE2L6* | -0.79 | -0.44 | 0.05 | 0.40 | 2 |
| *TNFSF10* | -0.73 | -0.55 | 0.01 | -0.33 | 2 |
| *ZCCHC2* | -0.96 | -0.42 | 0.04 | 0.12 | 2 |
| *SAMD9* | -0.62 | -0.07 | -0.14 | 0.69 | 2 |
| *TRIM25* | 0.91 | -0.20 | 0.02 | 0.31 | 2 |
| *CASP1* | -0.64 | -0.26 | -0.38 | 0.11 | 2 |
| *DHX58* | -0.74 | -0.39 | -0.21 | 0.06 | 2 |
| *PLSCR1* | -0.99 | -0.28 | -0.02 | -0.07 | 2 |
| *TMEM140* | -0.49 | -0.44 | 0.01 | 0.38 | 2 |
| *B2M* | -0.48 | -0.13 | 0.21 | 0.22 | 2 |
| *TRIM14* | -0.52 | -0.17 | 0.06 | 0.18 | 2 |
| *SP100* | -0.24 | -0.24 | 0.21 | 0.20 | 2 |
| *PPP1R11* | 0.29 | 0.41 | -0.03 | 0.07 | 2 |

* Number of cohorts in which the gene reached statistical significance (adj.P.Val < 0.05). adj.P.Val, adjusted P value (Benjamini–Hochberg correction); logFC, log_2_ fold change; N/F, not detected in the cohort.

**Table S7.** Interferon-related and immune genes significantly dysregulated in exactly 1 of 4 HCC transcriptomic cohorts among genes upregulated following *SMG7* depletion in SNU-387 cells (*n* = 31). logFC, log_2_ fold change.

| **Gene** | **TCGA logFC** | **GSE14520 logFC** | **GSE164760 logFC** | **GSE6764 logFC** | **Sig. cohorts*** |
| --- | --- | --- | --- | --- | --- |
| *IFI27* | 0.49 | -0.20 | 0.51 | 3.89 | 1 |
| *ANGPTL1* | -2.90 | N/F | 0.00 | -0.84 | 1 |
| *IFI6* | -0.35 | -0.02 | -0.09 | 2.85 | 1 |
| *CMPK2* | -0.61 | N/F | -0.07 | 1.48 | 1 |
| *MX1* | -0.38 | -0.12 | 0.02 | 2.27 | 1 |
| *RSAD2* | -0.97 | -0.04 | 0.05 | 1.25 | 1 |
| *GNB4* | -0.91 | N/F | -0.27 | -0.42 | 1 |
| *GBP5* | -0.68 | N/F | -0.05 | 0.80 | 1 |
| *DDX60L* | -0.81 | N/F | -0.17 | 0.55 | 1 |
| *TRIM69* | -0.30 | N/F | 0.00 | 1.08 | 1 |
| *TNFSF13B* | -0.81 | N/F | -0.09 | 0.38 | 1 |
| *USP18* | -0.79 | 0.04 | -0.01 | 0.85 | 1 |
| *IFI35* | 0.15 | 0.61 | -0.09 | 0.73 | 1 |
| *GBP4* | -0.64 | N/F | -0.08 | 0.44 | 1 |
| *PARP9* | -0.61 | N/F | -0.06 | 0.47 | 1 |
| *BATF2* | -0.31 | N/F | 0.11 | 0.68 | 1 |
| *SMDT1* | -0.14 | N/F | -0.05 | -0.80 | 1 |
| *MAP3K2* | -0.76 | -0.03 | 0.20 | 0.29 | 1 |
| *IFIH1* | -0.41 | -0.09 | -0.02 | 0.77 | 1 |
| *THEMIS2* | N/F | -0.33 | N/F | -0.31 | 1 |
| *ZNFX1* | -0.08 | N/F | -0.05 | 0.79 | 1 |
| *IFIT5* | -0.49 | 0.06 | -0.09 | 0.57 | 1 |
| *RIGI* | -0.38 | 0.06 | 0.04 | 0.72 | 1 |
| *JAGN1* | -0.41 | N/F | 0.09 | -0.34 | 1 |
| *STAT2* | -0.07 | -0.26 | -0.19 | 0.46 | 1 |
| *PNPT1* | 0.15 | N/F | 0.04 | 0.50 | 1 |
| *EIF2AK2* | -0.07 | 0.24 | 0.05 | 0.52 | 1 |
| *ETV7* | -0.06 | -0.11 | -0.05 | 0.62 | 1 |
| *SOCS1* | -0.38 | 0.20 | -0.03 | -0.16 | 1 |
| *ZBP1* | -0.39 | -0.09 | -0.15 | 0.14 | 1 |
| *SLC15A3* | -0.55 | -0.03 | -0.00 | 0.16 | 1 |

* Number of cohorts in which the gene reached statistical significance (adj.P.Val < 0.05). adj.P.Val, adjusted P value (Benjamini–Hochberg correction); logFC, log2 fold change; N/F, not detected in the cohort.

**Table S8.** Interferon-related and immune genes not reaching statistical significance in any of the 4 HCC transcriptomic cohorts, among genes upregulated following *SMG7* depletion in SNU-387 cells (*n* = 16). logFC, log_2_ fold change.

| **Gene** | **TCGA logFC** | **GSE14520 logFC** | **GSE164760 logFC** | **GSE6764 logFC** | **Sig. cohorts*** |
| --- | --- | --- | --- | --- | --- |
| *SAMD9L* | -0.24 | N/F | 0.06 | 0.92 | 0 |
| *MX2* | -0.11 | 0.16 | -0.15 | 1.01 | 0 |
| *PARP14* | -0.15 | N/F | 0.14 | 0.53 | 0 |
| *PARP10* | 0.26 | N/F | 0.13 | 0.39 | 0 |
| *GBP3* | -0.30 | N/F | -0.02 | 0.39 | 0 |
| *SP110* | -0.14 | 0.02 | -0.28 | 0.44 | 0 |
| *SYNC* | 0.75 | 0.01 | 0.01 | 0.10 | 0 |
| *SEC16B* | -0.24 | N/F | -0.08 | 0.27 | 0 |
| *HELZ2* | N/F | N/F | N/F | 0.17 | 0 |
| *TRIM21* | -0.17 | -0.03 | -0.12 | 0.31 | 0 |
| *NKRF* | 0.07 | 0.02 | 0.09 | 0.36 | 0 |
| *TMEM229B* | 0.24 | N/F | -0.04 | 0.09 | 0 |
| *TTC21A* | 0.23 | N/F | 0.01 | 0.10 | 0 |
| *LGALS9* | 0.03 | 0.09 | -0.19 | 0.13 | 0 |
| *MANBA* | -0.15 | -0.05 | 0.10 | 0.11 | 0 |
| *PRSS1* | N/F | N/F | -0.10 | N/F | 0 |

* Number of cohorts in which the gene reached statistical significance (adj.P.Val < 0.05). adj.P.Val, adjusted P value (Benjamini–Hochberg correction); logFC, log_2_ fold change; N/F, not detected in the cohort.

**Table S9.** Oligonucleotide primers used for quantitative reverse transcription PCR (RT-qPCR) and PCR-based expression arrays. All primers were designed using Primer-BLAST (NCBI) and span exon–exon junctions unless otherwise noted. Sequences are listed in the 5′→3′ direction.

| **Gene** | **Species** | **Forward primer (5′→3′)** | **Reverse primer (5′→3′)** |
| --- | --- | --- | --- |
| *RBM8A* | *Homo sapiens* | GATTATGACAGCGTGGAGCA | GTCTTCTTCGGTGGCTTCCT |
| *SRRM1* | *Homo sapiens* | GTAGCCCAAGAAGACGCAAA | TGGTTCTGTGACGGGGAG |
| *ALY/REF* | *Homo sapiens* | CTACAGCAGGCCAAAACAACT | AGTTTCCCACCTGTCTCCAC |
| *UPF1* | *Homo sapiens* | CTGCACACCAAGCTCTACCA | CACACAGGACAGGATGATGAA |
| *UPF2* | *Homo sapiens* | CAATGAACGGCAAGAACAAG | CTCCCTTCGGATGTTGGTAG |
| *UPF3A* | *Homo sapiens* | ACAGTCCAGCACCCAGAAAA | CTGTTTCCGCCACACTCTC |
| *ETF1* | *Homo sapiens* | GCACACTCCAAGGAAACACA | AAAACGCAAGGCTGACTGAC |
| *GSPT1* | *Homo sapiens* | ACCTTTTCTTACTGGCATGGA | ACTGCGTATTTTCCCCTTGA |
| *SMG1* | *Homo sapiens* | GAGCCAAAGAGCAAGTCAGG | AGTTCAAATAACGCCGCATC |
| *CBP80* | *Homo sapiens* | TGAAAGTCCCAAACCGAAGT | GCAGGACACAGAGCTGAGAA |
| *CBP20* | *Homo sapiens* | CGTCTGGATGACCGAATCA | TCGTAGTCCTGCCGATACTCA |
| *SMG8* | *Homo sapiens* | ACCAACAGGGCTTTATTCCA | AGCAGGCCAAGAGTTTGTGT |
| *SMG9* | *Homo sapiens* | ATTGCTGCCTTCCTTTTCAC | GGCTTCACCATCTCTGCTGT |
| *DHX34* | *Homo sapiens* | AAACCGCCATCCTCTACCTC | CAGCATCTTCCCAATCACAA |
| *SMG6* | *Homo sapiens* | CTGACTGCAAAAGGGTCACA | GCCACTGACACATACTTTCCAC |
| *DCP1A* | *Homo sapiens* | AGACAGCAGCAGCAAGAGTG | AGGCTGCACAAATACTTCAGG |
| *DCP2* | *Homo sapiens* | TATGACCCCCAAATCCAAAC | AATCTTCGAGAAAGCCAGTCC |
| *SMG5* | *Homo sapiens* | CCCCTCATAGGATGCAAGAA | GGACAAATCCCCCAGATACA |
| *SMG7* | *Homo sapiens* | ATGAAACCGAGCAGCACAC | GCACAGGATGCCAAGAAAA |
| *XRN1* | *Homo sapiens* | AGATTTTGATCGGGAGCACTT | CTTCTTCTGCTGCGACACCT |
| *PABPC1* | *Homo sapiens* | AGTTCGCAATCCTCAGCAAC | CCAACATGGAAGCAGTCAAA |
| *RNPS1* | *Homo sapiens* | TCTTCTGGCTCTCCAAGTCC | CGCCTTTTCCTCTCCTTTTC |
| *GADD45A* | *Homo sapiens* | GGAGGAATTCTCGGCTGGAG | CGTTATCGGGGTCGACGTT |
| *GADD45B* | *Homo sapiens* | TTGTCTCCTGGTCACGAACC | TGTGGCAGCAACTCAACAGA |
| *ATF3* | *Homo sapiens* | CCTCTGCGCTGGAATCAGTC | TTCTTTCTCGTCGCCTCTTTTT |
| *ZBP1* | *Homo sapiens* | AGCACTGGGAATGAGGACAG | CTTCTGGGCGGTAAATCGT |
| *ZBP1 (pre-mRNA)*^a^ | *Homo sapiens* | GAGCCCCGGAGAAGTTAATC | CCGGTAGTTGCAAACTCAGAC |
| *NLRP3* | *Homo sapiens* | CTGAAACAGCAGATGGAGAGTG | GCAGCAAACTGGAAAGGAAG |
| *IL1B* | *Homo sapiens* | TGGCAATGAGGATGACTTGTT | TGGTGGTGCGAGATTCGT |
| *MIF* | *Homo sapiens* | AGAACCGCTCCTACAGCAAG | CCGCGTTCATGTCGTAATAGT |
| β-actin | *Homo sapiens* | ACTCTTCCAGCCTTCCTTCCT | CAGTGATCTCCTTCTGCATCCT |
| *HPRT1* | *Homo sapiens* | TGCTGAGGATTTGGAAAGGGT | AGAGGGCTACAATGTGATGGC |
| *Smg7* | *Mus musculus* | GCTCCAAGGCAGAACTGAAC | GCTGGTGTCGAATGATCTGA |

^a^ Primer pair targeting the unspliced pre-mRNA of *ZBP1* to quantify nascent transcription independently of mature mRNA levels.

**Supplementary Figures**

**Figure S1. *SMG7* expression changes across MASLD-MASH-HCC stages in vitro models. (a)** Schematic of the MASLD *in vitro* model using free fatty acids (FFAs)-treated THLE-2 cells. Created with BioRender.com (agreement number: VI2A1QBT3Y) **(b-d)** mRNA expression of *NLRP3***,** *IL1B*, and *MIF* in THLE-2 cells exposed to FFAs**. (e)** *SMG7* mRNA expression in THLE2 cells treated with oleate (OA), palmitate (PA), or a combination (OA+PA). **(f)** *Smg7* mRNA expression in mouse primary hepatocytes (MPH) treated with palmitic acid (PA), and representative images of BODIPY and DAPI staining in control vs. PA-treated cells. **(g)** SMG7 protein levels in THLE-2, Hep3B and SNU-387 cell lines, **normalized to β-tubulin and expressed as % of control**. **mRNA expression values are shown as copy numbers normalized to the normalization factor (NF) and calculated as percentage (%) of control (control = 100%).** Protein levels are normalized to β-tubulin. Data are represented as mean ± standard error of the mean (SEM). Asterisks denote statistical significance (*p < 0.05; **p < 0.01; ***p < 0.001; ****p < 0.0001). The values displayed on the graphs correspond to p-values.

**Figure S2. Altered expression of *UPF1* under *SMG7* modulation in HCC cell lines.** UPF1 mRNA and protein levels, and phosphorylated UPF1 (p-UPF1) expression when *SMG7* is overexpressed in Hep3B **(a and b)** and SNU-387 **(c and d).** UPF1 mRNA and protein expression, and p-UPF1 levels after *SMG7* silencing in Hep3B **(e and f)** and SNU-387 **(g and h)**. mRNA expression values are calculated as copy numbers normalized to the normalization factor (NF) and percentage (%) of control (control = 100%). Protein levels are normalized to β-tubulin and expressed as % of control. Data is represented as mean ± SEM. Asterisks (* p <0.05; ** p <0.01) indicate statistically significant differences. The values displayed on the graphs correspond to p-values.

**Figure S3. Functional effects of *UPF1* downregulation in HCC cell lines.** Validation of *UPF1* knockdown (UPF1^KD^) **(a)** and SMG7 expression in Hep3B **(b)** and SNU-387 **(c** and **d)** at the mRNA and protein levels. **(e-l)** Cellular proliferation, colonies, cell migration, and tumorsphere formation of Hep3B and SNU-387 after *UPF1* silencing compared with the control (SCR). Evaluation of NMD efficiency by quantifying NMD target transcripts and p-UPF1 protein in Hep3B **(m and n)** and SNU-387 **(o and p)**. Data is presented as mean ± SEM. The sample size included at least three biological replicates. mRNA expression values are shown as copy numbers normalized to the normalization factor (NF) and calculated as percentage (%) of control (control = 100%). Protein levels are normalized to β-tubulin and expressed as % of control. Asterisks (* p<0.05; ** p<0.01; *** p<0.001) indicate statistically significant differences.

**Figure S4. Direct comparison of the phenotypic effects induced by *SMG7*, *UPF1*, and combined *SMG7/UPF1* knockdown.** Cellular proliferation was assessed in Hep3B (**a**) and SNU-387 (**b**) cells. Migration and colony number were assessed in Hep3B (**c**, **d**), and SNU-387 (**e**, **f**) cells, respectively. Tumorsphere number and mean tumorsphere size were assesed in Hep3B (**g**, **h**) and SNU-387 (**i**, **j**) cells, respectively, following *SMG7*, *UPF1*, or combined *SMG7/UPF1* silencing. Comparisons were performed against the control condition (SCR) and among knockdown conditions. Data are presented as mean ± SEM. The sample size included at least three biological replicates. Asterisks (* p < 0.05; ** p < 0.01; *** p < 0.001) indicate statistically significant differences relative to control (Student’s t-test), whereas # denotes statistically significant differences between knockdown conditions (one-way ANOVA).

**Figure S5. Differential effects on tumor weight after *SMG7* and/or *UPF1* silencing and validation of SMG7 and UPF1 protein levels. (a)** Tumor weight (% of control) in SMG7^KD^, UPF1^KD^ and SMG7^KD^UPF1^KD^ tumors at the endpoint. **(b)** SMG7 protein levels in SMG7^KD^ and SMG7^KD^UPF1^KD^ tumors at the endpoint. **(c)** UPF1 protein levels in UPF1^KD^ and SMG7^KD^UPF1^KD^ tumors at the endpoint. Protein levels are normalized to β-tubulin and expressed as % of control. Data are represented as mean ± SEM. The sample size included three biological replicates. P < 0.05 indicate statistically significant differences.

**Figure S6. Protein-protein interaction analysis and** **molecular functions of genes upregulated in SNU-387 cells upon *SMG7* knockdown. (a)** Search Tool for the Retrieval of Interacting Genes/Proteins (STRING) network analysis of genes upregulated after *SMG7* silencing in SNU-387, with colored nodes based on molecular function enrichment (Gene Ontology). **(b)** Diagram obtained from STRING representing molecular function enrichment (Gene Ontology) of 10 terms with FDR ≤ 0.05 and signal > 0.5 based on the upregulated genes in RNA-seq.

**Figure S7. WGCNA network construction, module expression trajectories, and key feature selection. (a)** Analysis of the scale-free topology fit index (signed R^2^) as a function of the soft-thresholding power (β). The red horizontal line indicates the threshold used to establish a scale-free topology. **(b)** Analysis of the mean connectivity (degree) versus the soft-thresholding power (β). Together with panel (a), β = 6 was selected as the optimal power for network construction. **(c)** Individual gene expression trajectories across samples partitioned by the six identified co-expression modules (Blue, Brown, Green, Grey, Turquoise, and Yellow). Colored lines represent individual gene expression levels (VST, Variance Stabilizing Transformation), and the solid black line represents the overall module expression trend across samples. **(d)** Variable Importance in Projection (VIP) score plot highlighting top-ranking genes from all modules. The heatmap on the right indicates the relative expression levels (High to Low) across different sample groups (NT: Non-Tumoral, RT: Recurrent Tumor, PT: Primary Tumor).

**Figure S8. Gene co-expression network generated from WGCNA and pathway enrichment analysis of module-associated genes. Nodes represent genes and are colored according to their module assignment. Node size reflects the number of connections (degree), with larger nodes indicating highly connected hub genes. Edges represent co-expression relationships between gene pairs, and their thickness is proportional to the correlation coefficient, with thicker edges indicating stronger correlations. Node colors correspond to the WGCNA modules identified in Fig. 7 and Fig. S7. Enriched pathways for each module are summarized in boxes colored according to the corresponding module. Network visualization and pathway enrichment analyses were performed using Cytoscape.**

**Supplementary references**

1. The ICGC/TCGA Pan-Cancer Analysis of Whole Genomes Consortium.  Pan-cancer analysis of whole genomes. Nature. 2020 Feb 6;578(7793):82–93. https://doi.org/10.1038/s41586-020-1969-6

2. Wurmbach E, Chen Y, Khitrov G, Zhang W, Roayaie S, Schwartz M, et al. Genome-wide molecular profiles of HCV-induced dysplasia and hepatocellular carcinoma†. Hepatology. 2007;45(4):938–47. https://doi.org/10.1002/hep.21622

3. Roessler S, Jia H-L, Budhu A, Forgues M, Ye Q-H, Lee J-S, et al. A Unique Metastasis Gene Signature Enables Prediction of Tumor Relapse in Early-Stage Hepatocellular Carcinoma Patients. Cancer Res. 2010;70(24):10202–12. https://doi.org/10.1158/0008-5472.CAN-10-2607

4. Pinyol R, Torrecilla S, Wang H, Montironi C, Piqué-Gili M, Torres-Martin M, et al. Molecular characterisation of hepatocellular carcinoma in patients with non-alcoholic steatohepatitis. J Hepatol. 2021;75(4):865–78. https://doi.org/10.1016/j.jhep.2021.04.049

5. Govaere O, Cockell S, Tiniakos D, Queen R, Younes R, Vacca M, et al. Transcriptomic profiling across the nonalcoholic fatty liver disease spectrum reveals gene signatures for steatohepatitis and fibrosis. Sci Transl Med. 2020;12(572):eaba4448. https://doi.org/10.1126/scitranslmed.aba4448

6. Gao Q, Zhu H, Dong L, Shi W, Chen R, Song Z, et al. Integrated Proteogenomic Characterization of HBV-Related Hepatocellular Carcinoma. Cell. 2019;179(2):561-577.e22. https://doi.org/10.1016/j.cell.2019.08.052

7. López-Cánovas JL, Del Rio-Moreno M, García-Fernandez H, Jiménez-Vacas JM, Moreno-Montilla MT, Sánchez-Frias ME, et al. Splicing factor SF3B1 is overexpressed and implicated in the aggressiveness and survival of hepatocellular carcinoma. Cancer Lett. 2021;496:72–83. https://doi.org/10.1016/j.canlet.2020.10.010

8. López-Cánovas JL, Hermán-Sánchez N, Moreno-Montilla MT, Del Rio-Moreno M, Alors-Perez E, Sánchez-Frias ME, et al. Spliceosomal profiling identifies EIF4A3 as a novel oncogene in hepatocellular carcinoma acting through the modulation of FGFR4 splicing. Clin Transl Med. 2022;12(11):e1102. https://doi.org/10.1002/ctm2.1102

9. Gahete MD, Del Rio-Moreno M, Camargo A, Alcala-Diaz JF, Alors-Perez E, Delgado-Lista J, et al. Changes in Splicing Machinery Components Influence, Precede, and Early Predict the Development of Type 2 Diabetes: From the CORDIOPREV Study. EBioMedicine. 2018;37:356–65. https://doi.org/10.1016/j.ebiom.2018.10.056

10. Bustin SA, Benes V, Garson JA, Hellemans J, Huggett J, Kubista M, et al. The MIQE Guidelines: Minimum Information for Publication of Quantitative Real-Time PCR Experiments. Clin Chem. 2009;55(4):611–22. https://doi.org/10.1373/clinchem.2008.112797

11. Lee SM, Pusec CM, Norris GH, De Jesus A, Diaz-Ruiz A, Muratalla J, et al. Hepatocyte-specific loss of PPARγ protects mice from NASH and increases the therapeutic effects of rosiglitazone in the liver. Cell Mol Gastroenterol Hepatol. 2021;11(5):1291–311. https://doi.org/10.1016/j.jcmgh.2021.01.003

12. Boehm V, Kueckelmann S, Gerbracht JV, Kallabis S, Britto-Borges T, Altmüller J, et al. SMG5-SMG7 authorize nonsense-mediated mRNA decay by enabling SMG6 endonucleolytic activity. Nat Commun. 2021;12:3965. https://doi.org/10.1038/s41467-021-24046-3

13. Kurosaki T, Li W, Hoque M, Popp MW-L, Ermolenko DN, Tian B, et al. A post-translational regulatory switch on UPF1 controls targeted mRNA degradation. Genes Dev. 2014;28(17):1900–16. https://doi.org/10.1101/gad.245506.114

14. Andrews S. FastQC: a quality control tool for highthroughput sequence data. 2010. Available from: https://www.bioinformatics.babraham.ac.uk/projects/fastqc/

15. Krueger F. Trim Galore: a wrapper tool around Cutadapt and FastQC to consistently apply quality and adapter trimming to FastQ files. 2012. Available from:  https://www.bioinformatics.babraham.ac.uk/projects/trim_galore/

16. Patro R, Duggal G, Love MI, Irizarry RA, Kingsford C. Salmon provides fast and bias-aware quantification of transcript expression. Nat Methods. 2017;14(4):417–9. https://doi.org/10.1038/nmeth.4197

17. Soneson C, Love MI, Robinson MD. Differential analyses for RNA-seq: transcript-level estimates improve gene-level inferences. 2016. https://doi.org/10.12688/f1000research.7563.2

18. Love MI, Huber W, Anders S. Moderated estimation of fold change and dispersion for RNA-seq data with DESeq2. Genome Biol. 2014;15(12):550. https://doi.org/10.1186/s13059-014-0550-8

19. Subramanian A, Tamayo P, Mootha VK, Mukherjee S, Ebert BL, Gillette MA, et al. Gene set enrichment analysis: A knowledge-based approach for interpreting genome-wide expression profiles. Proc Natl Acad Sci U S A. 2005;102(43):15545–50. https://doi.org/10.1073/pnas.0506580102

20. GenePattern Platform. Gene Set Enrichment Analysis (GSEA) and ssGSEA modules. Available from: https://www.genepattern.org/

21. R Core Team. R: A language and environment for statistical computing. 2023. Available from: https://www.r-project.org/

22. Langfelder P, Horvath S. WGCNA: an R package for weighted correlation network analysis. BMC Bioinformatics. 2008;9(1):559. https://doi.org/10.1186/1471-2105-9-559

23. Shannon P, Markiel A, Ozier O, Baliga NS, Wang JT, Ramage D, et al. Cytoscape: A Software Environment for Integrated Models of Biomolecular Interaction Networks. Genome Res. 2003;13(11):2498–504. https://doi.org/10.1101/gr.1239303

24. Raudvere U, Kolberg L, Kuzmin I, Arak T, Adler P, Peterson H, et al. g:Profiler: a web server for functional enrichment analysis and conversions of gene lists (2019 update). Nucleic Acids Res. 2019;47(W1):W191–8. https://doi.org/10.1093/nar/gkz369

25. Boehm V, Wallmeroth D, Wulf PO, Popp O, Teixeira Alves LG, Reinecke L, et al. Rapid UPF1 depletion illuminates the temporal dynamics of the NMD-regulated human transcriptome. Mol Cell. 2025;85(18):3524-3546.e12. https://doi.org/10.1016/j.molcel.2025.08.015
